# Development of a *Bmi1*+ Cardiac Mouse Progenitor Immortalized Model to Unravel the Relationship with Its Protective Vascular Endothelial Niche

**DOI:** 10.3390/ijms25168815

**Published:** 2024-08-13

**Authors:** Guillermo Albericio, Marina Higuera, Paula Araque, Cristina Sánchez, Diego Herrero, Miguel A. García-Brenes, Laura Formentini, José Luis Torán, Carmen Mora, Antonio Bernad

**Affiliations:** 1 Cardiac Stem Cells Lab, Immunology and Oncology Department, National Center for Biotechnology (CNB-CSIC), Campus Universidad Autónoma de Madrid, 28049 Madrid, Spain; galbericio@cnb.csic.es (G.A.); mhiguera@cnb.csic.es (M.H.); paulaaraque28@gmail.com (P.A.); jltoran@cnb.csic.es (J.L.T.);; 2 Molecular Biology Department, National Center for Biotechnology (CNB-CSIC), Campus Universidad Autónoma de Madrid, 28049 Madrid, Spain; 3 Molecular Biology Department, Molecular Biology Center Severo Ochoa (CBMSO), Campus Universidad Autónoma de Madrid, 28049 Madrid, Spain

**Keywords:** heart, progenitor, *Bmi1*, immortalization, *Bmi1*-DR, SV40-T, endothelial, niche, oxidative damage, senescence, autophagy, metabolism

## Abstract

The adult mammalian heart has been demonstrated to be endowed with low but real turnover capacity, especially for cardiomyocytes, the key functional cell type. The source, however, of that turnover capacity remains controversial. In this regard, we have defined and characterized a resident multipotent cardiac mouse progenitor population, *Bmi1*+DR (for *Bmi1*+ Damage-Responsive cells). *Bmi1*+DR is one of the cell types with the lowest ROS (Reactive Oxygen Species) levels in the adult heart, being particularly characterized by their close relationship with cardiac vessels, most probably involved in the regulation of proliferation/maintenance of *Bmi1*+DR. This was proposed to work as their endothelial niche. Due to the scarcity of *Bmi1*+DR cells in the adult mouse heart, we have generated an immortalization/dis-immortalization model using Simian Vacuolating Virus 40-Large Antigen T (SV40-T) to facilitate their in vitro characterization. We have obtained a heterogeneous population of immortalized *Bmi1*+DR cells (*Bmi1*+DR^IMM^) that was validated attending to different criteria, also showing a comparable sensitivity to strong oxidative damage. Then, we concluded that the *Bmi1*-DR^IMM^ population is an appropriate model for primary *Bmi1*+DR in vitro studies. The co-culture of *Bmi1*+DR^IMM^ cells with endothelial cells protects them against oxidative damage, showing a moderate depletion in non-canonical autophagy and also contributing with a modest metabolic regulation.

## 1. Introduction

The notion of the mammalian adult heart as a terminally differentiated organ has been challenged during the last 20 years [1]. Cardiomyocyte (CM) proliferation has been demonstrated in young individuals (<20 years old) [2] and upregulated in some pathological conditions, like myocardial infarction (MI) [3]. Despite different hypotheses for cell turnover being explored in cardiac tissue, the source(s) of new cells in the adult mammalian heart remain enigmatic (reviewed by [4]). Cardiomyocyte turnover has been proposed, being sustained by the de-differentiation/proliferation/re-differentiation of certain mature mouse CM; however, this proposal has demonstrated a very low frequency (<1% of annual turnover) and still awaits more robust empirical verification [5].

Tissue homeostasis, repair, and damage response mainly relies on the regulated activity of scarce populations of tissue-specific adult stem cells (ASCs)/progenitors. Classical studies on ASCs have relied on the use of supposed specific ASC markers and tracing of their progeny. In tissues with a high cell turnover, these ASC populations are clearly defined (reviewed by [6,7,8]). Regarding the adult heart, several markers have been proposed for the identification/isolation of cardiac resident stem/progenitor cells (multipotent, CSC) [9]. Among them, *c-Kit*^+^ cardiac cells were the first and the most intensively studied candidate population [10,11] as a potential resource for cardiovascular therapy. However, later findings from basic and preclinical research, together with the failure of several clinical trial evaluations, have fueled a long and bitter debate over the relevance of *c-Kit*^+^ CSC [12]. Other progenitor-like cell populations have been described, most of these proposals being based on cell surface proteins that are also expressed on other ASCs or markers associated with general progenitor functions, such as *Sca1*, *Abcg2*, or *Isl1* (reviewed in [13]). As an independent strategy to define elusive CSC, expression of *Bmi1* as the most representative marker of mouse adult stem cell compartments (reviewed by [14]) was evaluated in the adult mouse heart.

*BMI1* is a member of the Polycomb Repressive Complexes 1 (PRC1), a well-recognized transcriptional suppressor with the ability to drive self-renewal and proliferation of many tissue-specific stem cells [15]. Using appropriated mouse models, we confirmed the existence of a non-cardiomyocyte *Bmi1*+ population; around 75,000 *Bmi1+* cells were estimated in the mouse adult heart [15]. *Bmi1*+DR cells contain two major, and mutually exclusive, subpopulations: PDGFRα+ CD31- and CD31+ PDGFRα- populations. In previous studies, we have demonstrated the in vitro and in vivo multilineage cardiac differentiation capacity of *Bmi1+*DR cells, showing their contribution to the basal “wear and tear” of cardiac endothelial cell (EC), vascular smooth cell (SMC), and CM lineages, with a substantial endothelial bias both in homeostasis and in response to acute myocardial infarct (AMI) as well as other forms of cardiac damage [15,16,17,18,19]. Transcriptional analysis demonstrated that *Bmi1* expression is linked to a mixture of endothelial- and mesenchymal-related non-myocyte *Sca1+* cells in the adult mouse heart, although the level of expression of endothelial genes was significantly lower in *Bmi1+* cells when compared with mature endothelial cells [17,18,19].

Regarding tissue repair, solid evidence has demonstrated that cardiac *Bmi1*+ progenitors are quite resistant to several forms of damage, like AMI, instead becoming proliferatively activated, with a net increase in their cell numbers during the first few days [16,17,18]. Moreover, their progeny showed an enhanced contribution to the main de novo cell lineages, including CM, when compared with cells from age-paired non-infarcted adult hearts [18,20]. Due to this capacity, this population is called *Bmi1*+DR cells for *Bmi1*+ Damage-Responsive [19]. Redox status influences *Bmi1*+DR cells response and highlights redox-mediated BMI1 regulation, with implications for the maintenance of cellular identity in vivo [17]. It was estimated that at 4 months post-AMI, the *Bmi1*+DR population contributes up to 20% of total endothelial cells in the infarcted heart [18]. The relevant in vivo physiological role of the *Bmi1*+DR population has been validated by a genetic ablation model; when *Bmi1*+DR cell ablation was coupled to AMI, animals manifested signs of cardiac dysfunction, affecting the survival. Perimortem analysis revealed a dilated cardiomyopathy-like phenotype with a significant deficit in the angiogenic response to AMI as the most probable cause of death [18].

The ASC niche, defined as the local (micro)environment surrounding a stem cell-containing population, is now recognized as the functional unit for ASC maintenance and regulation [21]. Stem cell niches are dynamic functional domains rich in specialized cells that influence, condition, and coordinate ASC behavior to govern tissue homeostasis under physiological conditions, but in certain contexts, the stem cell niche can be corrupted, as in some cancers and chronic pathologies [22,23,24]. ASC niche might function both through direct cell–cell contact and by releasing soluble factors. However, recent studies on the long-term lympho-hematopoietic stem cell (HSC) niche, undoubtedly the best-characterized model of the ASC niches, have uncovered new layers of regulatory complexity. For instance, HSC progenies themselves regulate HSC behavior, lineage-biased differentiation of HSCs is coordinated by distinct niches, and HSCs can remodel their own perivascular niche [25,26,27]. Currently, based on niche composition, this well-described perivascular niche has been defined in other ASCs (e.g., neural stem cells, cancer stem cells) with a similar level of complexity. In any case, scarce research has been invested in the characterization of adult heart niches.

Aiming to identify and define the *Bmi1*+DR cell niche(s), we analyzed mouse adult heart sections to find that the majority of *Bmi1*+DR cells were located in the left ventricle (≈70%) in a gradient-like distribution around the cardiac vasculature, and preferentially close to small vessels [19]. Interestingly, in the adolescent heart and earlier ages, *Bmi1*+DR cells display an almost random distribution, suggesting that perivascular confinement becomes relevant in an age-dependent manner, with the progressive increasing of oxidative stress. Results confirmed that *Bmi1*+DR cells are sheltered in low-ROS perivascular domains, allowing us to propose that these structures form part of the perivascular niche for the *Bmi1*+DR population in the adult heart [19] (reviewed by [13]). Furthermore, in vivo evaluation of *Bmi1*+DR cells proliferative status relative to cardiac vasculature demonstrated that only a small percentage of these cells (≈10% of total) were proliferating. Equivalent analysis on infarcted hearts showed a strong distortion of the spatial distribution of *Bmi1*+DR cells in relation to cardiac vessels [19]. Altogether, these results showed that non-proliferating *Bmi1*+DR (quiescence-like) cells are preferentially located in strict areas close to the endothelium in homeostasis, strongly suggesting functional interactions. Preliminary evaluation [19] confirmed that, specifically, the co-culture of *Bmi1*+DR with endothelial cells decreases both of their proliferation rates, incrementing the expression of *Bmi1* as expected [28,29]. In direct contact, endothelial cells also promoted a reduction in total ROS, which was concomitant with a decrease in total mitochondrial mass, a hallmark of *Bmi1*+DR [19].

In conclusion, as in other ASC models, a perivascular niche for *Bmi1*+DR cells is envisioned. Better comprehension of the regulation of the cardiac niche(s) would be key for resolving uncertainties about the involvement of cardiac progenitor cells/stem cells in heart homeostasis and damage repair, and for demonstrating whether the low margin of heart turnover is relevant for healthy aging or counteracting some pathological scenarios. Then, in this study, we generated and characterized an in vitro *Bmi1*+DR immortalized cell model in an attempt to overcome some of the technical limitations associated with work involving such a scarce population of primary cells. Additionally, we applied this model to unravel some molecular mechanism(s) that could define the nature of this niche-like relationship among *Bmi1*+DR cells and the cardiac endothelium.

## 2. Results

### 2.1. Bmi1+DR Cells Correspond to a Vascular-Juxtaposed Population of Cardiac Progenitors Regulated by Oxidative Stress

**Figure 1:**
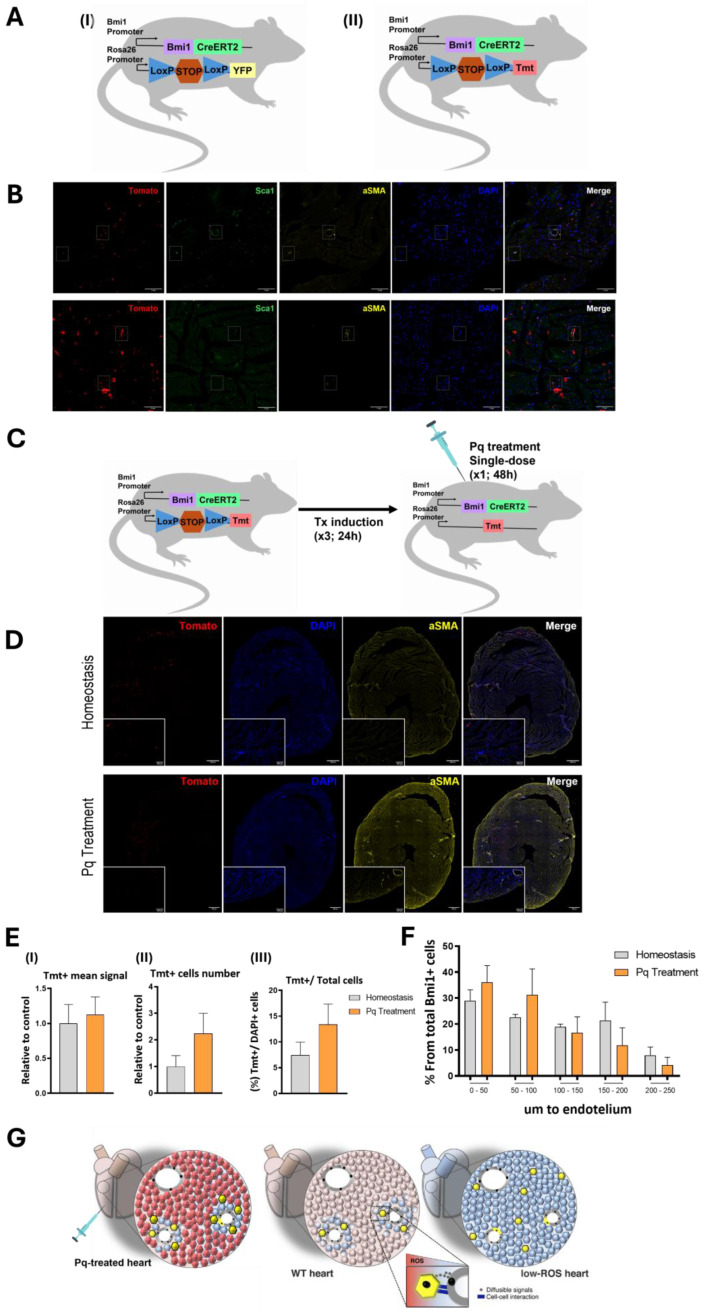
Endogenous *Bmi1*+DR population shows a clear perivascular location regulated by oxidative stress. (**A**) Linage tracing *Bmi1^CreERT/+^Rosa26^YFP/+^* (I) and *Bmi1^CreERT/+^Rosa26^tdTomato/+^* (II) mouse models. (**B**) Representative images showing double-positive *Tomato+ Sca1+ Bmi1*+DR cell juxtaposed localization to vascular structures (αSMA+) in *Bmi1^CreERT/+^Rosa26^tdTomato/+^* mice 5d post-Tx induction. Bars, 71 μm. (**C**) Scheme of in vivo single-dose Pq treatment (48h) in *Bmi1^CreERT/+^Rosa26^tdTomato^*^/+^ mice 5d post-Tx induction. (**D**) Total and partial (inset) mosaic (maps) images of transverse heart cryosections of single-dose Pq-treated (up) and non-treated *Bmi1*^CreERT/+^*Rosa26^tdTo^*^mato/+^ mice (down) showing *Tomato+ Bmi1*+DR cell number and localization with respect to vascular structures (αSMA+). Bars in partial and total mosaic images, 100 and 500 μm respectively. (**E**) Analysis of TOMATO mean signal (I), *Tomato+* cell numbers (II), and relativized total *Tomato+* cell (*Tomato+*/*Dapi*+) numbers (III) in single-dose Pq-treated (Pq Treatment) compared to non-treated (homeostasis) *Bmi1*^CreERT/+^*Rosa26^tdTomato/+^* mice on maps of transverse heart cryosections (*n* = 3; >1000 *Bmi1*+ cells/heart). (**F**) Distribution of *Bmi1*+DR cells in relation to coronary vasculature of single-dose Pq-treated (Pq Treatment) compared to non-treated (homeostasis) *Bmi1^CreERT/+^Rosa26^tdTomato/+^* mice on maps of transverse heart cryosections (*n* = 3; >1000 *Bmi1*+ cells/heart). (**G**) Graphical model of how oxidative stress affects *Bmi1*+ vascular niche. Low oxidative stress (Low-ROS G6PDtg mice) distorts *Bmi1*+DR cell localization on vascular structures, while high oxidative stress (Pq treatment) increases proximity and numbers.

*Bmi1*+DR (*Bmi1*+*Sca1*+; *Bmi1*+Damage-Responsive) cells were previously characterized in adult mice as a multilineage differentiation *Sca1*+ subpopulation with a marked endothelial bias both in homeostasis and in response to cardiac damage [15,18]. Figure S1A summarizes those typically considered endothelial preferent genes, comparing their level of expression in *Bmi1*+DR and *Bmi1*- *Sca1+* cells. Through suitable lineage tracing mouse models (Figure 1A, *Bmi1^CreERT/+^*-*Rosa26^YFP/+^*; *Bmi1^CreERT/+^*-*Rosa26^TdTomato^*^/+^), *Bmi1*+DR cells, as well their progeny, can be efficiently labelled with fluorescent reporter proteins (YFP or TOMATO) along the *Bmi1*+ lineage after Tx induction. As previously mentioned, based on *Bmi1*+DR cells vascular-juxtaposed distribution, an endothelial niche has been proposed. Using these mouse models, here we confirmed that in vivo, the perivascular *Bmi1*+DR population is highly enriched in double-positive *Bmi1*+*Sca1+* cells (Figure 1B). We marked the *Bmi1*+DR cells using Tx-inducible *Bmi1^CreERT/+^Rosa26^tdTomato/+^* mice (hereafter Bmi1-Tomato); additionally, *Sca1+* cells and vascular structures (αSMA; labelling vascular walls) were also labelled to confirm that the Bmi1*Tomato+ cells are among the *Bmi1*+DR population (*Bmi1+Sca1+)* and localized juxtaposed to the nearest endothelium.

Oxidative stress has been proposed as a key in vivo regulator of *Bmi1*+DR in homeostasis and responses upon damage [15,16,17]. *Bmi1*+DR cells in vivo were demonstrated to be quite resistant to cardiac damage as AMI, incrementing their contribution to the mature cell compartment (summarized in Figure S1B) [16]. Aiming to achieve a deeper analysis in this redox regulation in relation to their vascular niche, we questioned how this damage-associated ROS increase could affect this relationship. For externally inducing the ROS increase associated with cardiac damage, we treated Bmi1-Tomato mice with Paraquat (Pq), an herbicide that provokes superoxide radical formation employed both in in vitro and in vivo damage studies [30]. Previously, we validated that in Bmi1-Tomato mice at 5 days post-Tx induction, Pq treatment increases the cardiac ROS levels using CellROX staining [19]. So, for Pq treatment, we followed the same scheme. We administered Tx to adult Bmi1-Tomato mice and, after 5 days, a single-dose of Pq (20 mg/kg body weight; i.p.); then, 48 h after Pq treatment, animals were sacrificed and we proceeded to the cardiac immunofluorescence analysis to study the numbers and distribution of *Bmi1*+DR cells, comparing Pq treatment and homeostasis conditions (Figure 1C). In heart cryosections, *Bmi1*+DR cells were identified due to *Tomato+* reporter signal, DAPI staining allowed us to calculate an average of total cellularity per section (assuming one nuclei/cell), and vascular wall structures were labelled using anti-αSMA (Figure 1D). Differences between Pq treatment and homeostasis conditions were detected. First, we detected a slight increase in the mean signal of TOMATO reporter after Pq treatment (Figure 1E(I)) (*n* = 3, each; >1000 *Bmi1*+ cells/heart); to corroborate whether this difference came from a difference in the number of *Tomato+* cells, additional analyses were carried out to measure the number of *Tomato+* cells per section (Figure 1E(II)) and the percentage of *Tomato+* cells with respect to total cells (DAPI+) per section (Figure 1E(III)), resulting in an increase in both analyses in Pq-treated hearts when compared to control non-treated (homeostasis) hearts (*n* = 3, each; >1000 *Bmi1*+ cells/heart). Finally, using maps of transverse heart cryosections (Figure 1D), we analyzed the distribution of *Bmi1*+DR cells with respect to cardiac vasculature (αSMA labelling) (*n* = 3; >1000 *Bmi1*+ cells/heart) (Figure 1F); in this analysis, we observed the same trend as in control homeostasis conditions, with the endothelium relationship being even more remarkable, with a slight increase in the percentage of *Bmi1*+ (*Tomato+*) cells in proximity to vascular structures (0–50 μm, 50–100 μm) from total *Bmi1*+ cells and a decrease in the furthest ranges (150–200 μm, 200–250 μm). These results, despite not having statistical significance, show how high ROS levels in cardiac tissue affect the cellular state of the *Bmi1*+DR population, increasing their number, triggering their activation, and pointing to a tighter relationship with the endothelium in response to damage, unlike low-ROS conditions (glucose-6-phosphate dehydrogenase transgenic mice; G6PDTg), where we previously demonstrated that the niche-like structure became distorted [19] (Figure 1G). All of these data reinforce the vascular niche hypothesis for *Bmi1*+DR cells regulated by oxidative stress.

### 2.2. Generation and Characterization of a Conditionally Immortalized Bmi1+DR Population

**Figure 2:**
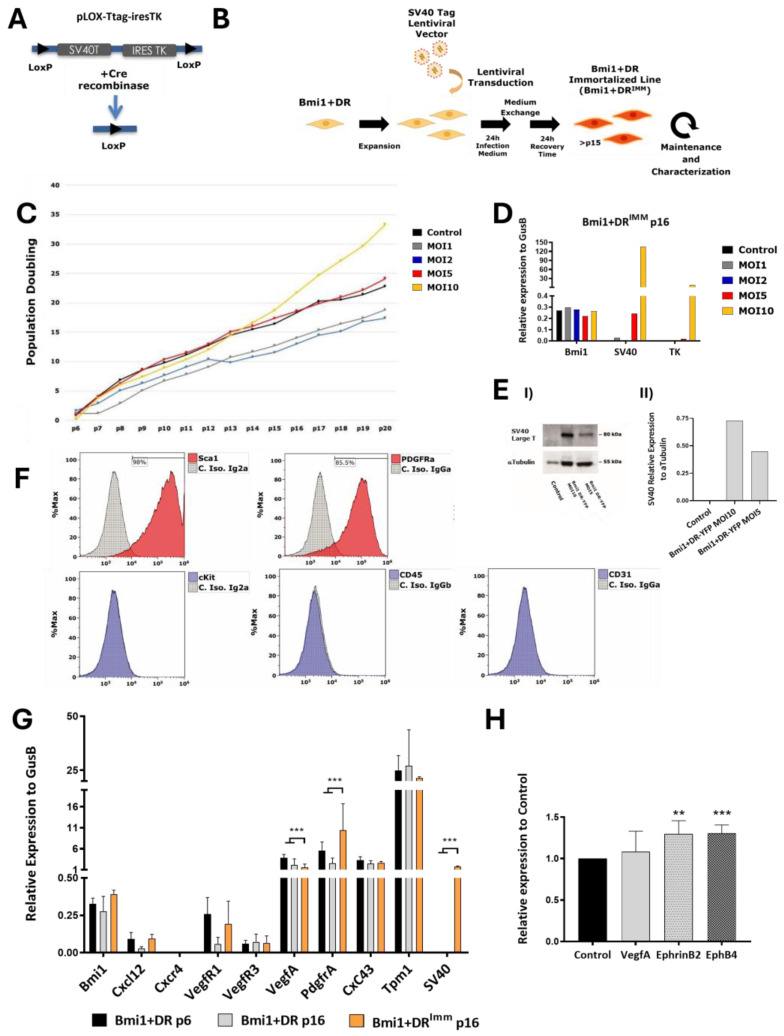
Generation and characterization of a conditionally immortalized *Bmi1*+DR population. (**A**) Representation of the SV40-T/TK immortalization vector used in the generation of the immortalized *Bmi1*+DR cell population. (**B**) Scheme of the procedure followed for the generation of the immortalized *Bmi1*+DR population through transduction of the SV40-T/TK lentiviral vector. (**C**) Cumulative population doubling rate (*Y* axis) after successive passes (p; *X* axis) of primary *Bmi1*+DR cells transduced with the different indicated MOIs. (**D**) Comparative SV40-T and TK expression by RT-qPCR analysis (relative to GusB) in primary *Bmi1*+DR cells treated with the different MOIs evaluated. (**E**) SV40-T protein expression by Western Blot in *Bmi1*+DR cells transduced with MOI 5 and 10 (I) and the corresponding analysis relative to αTUBULIN protein as control (II). (**F**) Evaluation of different membrane markers characterized by the *Bmi1*+DR population in the immortalized line by flow cytometry. (**G**) Comparative RT-qPCR analysis evaluating the expression relative to the endogenous *GusB* control of genes defining the *Bmi1*+DR population comparing early passage (p6) primary *Bmi1*+DR cells, control *Bmi1*+DR cells maintained during the immortalization process (p16), and final *Bmi1*+DR^IMM^ population (p16) (*n* = 3); SV40-T expression was evaluated as confirmation of immortalized nature. (**H**) *Bmi1* expression analysis by RT-qPCR after stimulation of *Bmi1*+DR^IMM^ cells with different recombinant proteins (VEGFA, EPHRINB2, EPHB4); untreated *Bmi1*+DR^IMM^ cells as control (*n* = 6). Statistical analyses: ** *p* < 0.01, *** *p* < 0.001; one-way ANOVA Bonferroni post-test.

Globally, and especially with minoritarian adult stem cell populations and their primary cultures, there are serious technical limitations, mainly with their scarce numbers (around 7.5 × 10^4^ *Bmi1*+DR cells/heart) and poor ex vivo culture conditions. This hampers many research options for the study of *Bmi1*+DR. In order to partially overcome this issue, we first aimed to develop a *Bmi1*+DR conditional immortal population (*Bmi1*+DR^IMM^) and confirmed that it would be a suitable model maintaining the main features of *Bmi1*^+^DR cells. For developing this model, we used SV40-T, previously demonstrated to be capable of immortalizing many cell lineages (reviewed by [31]) and, more recently, mouse cardiomyocyte progenitors [32]. Therefore, we used a reversible immortalization strategy (see Section 4.6.1) by the induction of the constitutive expression of SV40-T using lentiviral transduction (Figure 2A) of freshly isolated *Bmi1*+DR, obtained from tamoxifen *Bmi1^CreERT/+^Rosa26^YFP/+^* mice, 5 days post-Tx induction (Figure 1A; hereafter Bmi1-Cre^YFP^) or *Bmi1^GFP/+^* mice (Figure 1B and Figure S2A,B). After obtention and characterization, our dis-immortalization strategy is based on two LoxP sequences that were introduced in the immortalization lentiviral vector flanking the SV40-T/TK genes (Figure 2A). Then, the transient expression of Cre recombinase induced by an adenoviral vector would eliminate the immortalization cassette. In addition, there is a safety control in the lentiviral vector based on the constitutive co-expression of a negative selection gene (thymidine kinase; TK) to the SV40-T gene, encoded by the lentiviral vector. After this adenoviral Cre mediated dis-immortalization, ganciclovir administration should eliminate cells that were not deleted in the immortalization cassette (Figure 2A).

First, we isolated *Bmi1*+DR cells by FACS (Bmi1-Cre^YFP^) and we followed their replication capacity through accumulating population doublings (Figure 2C). In comparison with the other conditions, which, upon expansion, entered a stationary growth phase, *Bmi1*+DR cells transduced with MOI-10 were demonstrated to be immortalized, as they showed exponential growth; hereafter, we denote the immortalized pool as *Bmi1*+DR^IMM^. The expression of the immortalization cassette was evaluated by RT-qPCR and we found that *Bmi1*+DR cells transduced with MOI-10 showed a sustained expression of SV40-T and TK (Figure 2D). SV40-T expression was also confirmed by Western Blot of *Bmi1*+DR transduced with MOI 5 or 10 (Figure 2E; Figure S5).

An equivalent scheme was evaluated for *Bmi1*+DR cells from *Bmi1^GFP/+^* mice (Figure S2A) (hereafter, *Bmi1*+GFP cells), depicted in Figure S2B, but we did not obtain immortalized cells (Figure S2C). Although the immortalization/negative selection cassette is clearly expressed (Figure S2D,E), its level of expression declined during further culture (Figure S2F). Analysis of the presence of the immortalization cassette in the genomic DNA of *Bmi1*+GFP cells was part of the lentiviral transduction validation process. PCR reaction on the genomic DNA of cells was performed for the detection of the SV40 immortalization gene; we observed lentiviral transduction, but the integrated provirus seemed unstable, reducing their representation with culture passages, so a reduced capacity for NHEJ probably increases the level of non-integrated linear or circulated forms (Figure S2G). Most probably, this phenotype could be related to the haploinsufficiency for *Bmi1* (*Bmi1*+/−) (Figure S2A) in this model [33].

We then preliminarily tested whether the *Bmi1*+DR^IMM^ population maintains the main features defined for the primary *Bmi1*+DR population [15,19]. We first confirmed by flow cytometry that *Bmi1*+DR^IMM^ maintains a similar profile (SCA1^pos^, PDGFRα^pos^, c-KIT^neg^, CD45^neg^) for the main membrane molecules which define the *Bmi1*+DR population (Figure 2F). In addition, we evaluated, by RT-qPCR, whether some of the main genes expressed by *Bmi1*+DR cells at early and late expansion points (p6 or p16) maintain a similar expression profile in *Bmi1*+DR^IMM^, at equivalent passage (p16). Expression profiles between *Bmi1*+DR and *Bmi1*+DR^IMM^ were quite similar (Figure 2G), and interestingly, significant differences were only observed in *VegfA* and *Pdgfrα*. Some smaller differences (*Bmi1*, *VegfR3, Cxc43, Tpm1, Cxcl12,* and *VegfR1*) were found in *Bmi1*+DR^IMM^, indicating an expression level closer to the primary control *Bmi1*+DR cells at earlier (p6) passage. In conclusion, we have found that the *Bmi1*+DR^IMM^ population mainly corresponds to a primary *Bmi1*+DR subpopulation characterized by null expression of CD31 (protein) and higher levels of *Bmi1*. Finally, we evaluated the ability of the *Bmi1*+DR^IMM^ population to respond to physiological stimulus in a manner similar to that described in the original population. In primary *Bmi1*+DR cells, an increase in *Bmi1* expression has been shown after the exposure of signals derived from the endothelium, like VEGFA treatment in the culture medium or direct contact with signaling molecules like EPHRINB2 or EPHB4 [19]. We confirmed these responses in *Bmi1*+DR^IMM^ cells, although the *Bmi1* expression increase promoted by VEGFA treatment was modest, and not statistically significant (Figure 2H).

### 2.3. Bmi1^+^DR^IMM^ Dis-Immortalization Provokes a Sudden Senescent Phenotype

**Figure 3:**
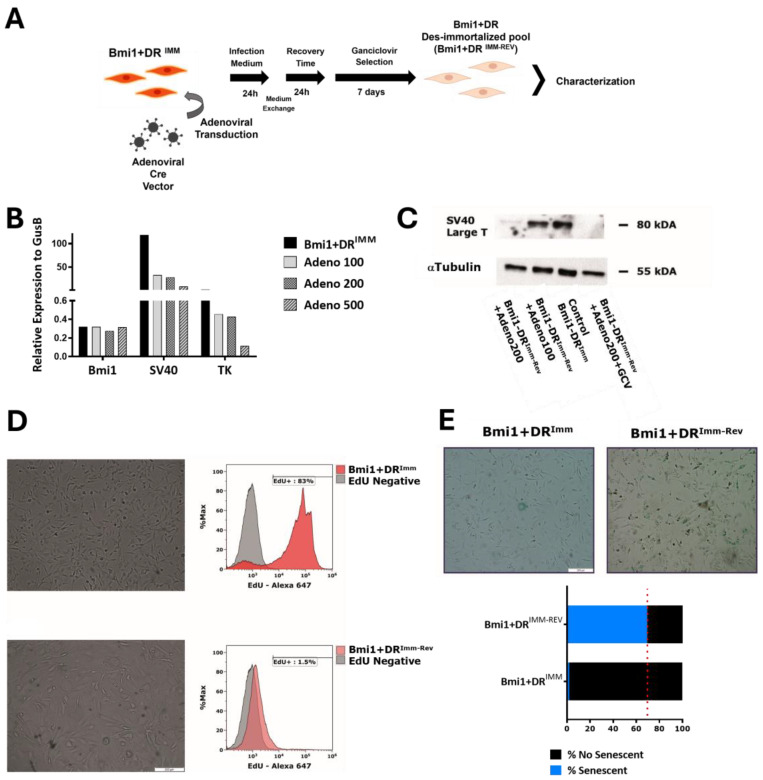
*Bmi1*+DR^IMM^ dis-immortalization provokes a sudden senescent phenotype. (**A**) Scheme of the procedure followed for *Bmi1*+DR^IMM^ cell dis-immortalization (*Bmi1*+DR^IMM-REV^) through transduction with an adenoviral vector that induces the expression of the Cre recombinase. (**B**) Comparative SV40-T and TK expression by RT-qPCR, relative to endogenous GusB gene expression, in *Bmi1*+DR^IMM^ cells treated in the dis-immortalization process with different MOIs of Cre adenoviral vector. (**C**) SV40-T protein expression by Western Blot in the *Bmi1*+DR^IMM^ cell line transduced in the process of dis-immortalization with different MOIs of Cre adenoviral vector and after negative selection with Ganciclovir (GCV). (**D**) Bright field representative images showing cellular morphology (left) and quantification of the percentage of proliferating cells by incorporating EdU for 12 h (right) in *Bmi1*+DR^IMM^ cell line before (up) and after (down) the dis-immortalization process. Scale bar, 200 μm. (**E**) β-galactosidase-based staining for senescent cells (blue color) comparing *Bmi1*+DR^IMM^ (left) and *Bmi1*+DR^IMM-REV^ (right) cells and the corresponding quantification of senescent cells observed per field in each of the cell types analyzed (red dot line highlighting difference between cell types). Scale bar, 200 μm.

Prior to using immortalized *Bmi1*+DR^IMM^ cells in in vitro studies, we evaluated the efficacy of the proposed dis-immortalized strategy planned for *Bmi1*+DR^IMM^. The immortalization cassette harbors TK joined to SV40-T expression, which, in combination with ganciclovir (GV), could be used in the recovery and enrichment of those cells that would have deleted the cassette, becoming dis-immortalized, as previously demonstrated [34]. Deletion of the immortalization cassette was accomplished using transduction with an adenovirus expressing Cre recombinase (Adeno-Cre) constitutively (Figure 3A) (see Section 4.6.4). Results clearly showed that there was a direct correlation between Adeno-Cre dose (MOI-100, -200, and -500) and reduction in the expression of the immortalization cassette (SV40-T and TK) both by RT-qPCR (Figure 3B) and Western Blot (Figure 3C; Figure S6). We selected the dose of MOI-200 of Adeno-Cre for further evaluations (Figure S3A) using *Bmi1*+DR^IMM^, and it was demonstrated by Western Blot that treatment with GV fully eliminates SV40-T (Figure 3C; Figure S6). However, dis-immortalized *Bmi1*+DR^IMM^ (called *Bmi1*+DR^IMM-REV^) showed important morphology alterations and a drastic restriction in proliferation, which prevents their expansion in culture. *Bmi1*+DR^IMM^ cultures showed 83% of EdU+ proliferative cells, in sharp contrast with *Bmi1*+DR^IMM-REV^ cultures, which showed around 1.5% of proliferating cells (Figure 3D); furthermore, morphology changes strongly suggested senescence development. Evaluation of β-galactosidase staining confirmed that *Bmi1*+DR^IMM-REV^ cultures presented a much higher level of β-gal+ cells (69.5%) in comparison with control *Bmi1*+DR^IMM^ cultures (around 2%) (Figure 3E). This scenario hampered the direct analysis of *Bmi1*+DR^IMM-REV^ populations. Then, we tried to explore alternative in vitro culture conditions to partially bypass some of the adverse effects imposed on the dis-immortalized cells using strategies previously described in other cell models [35,36,37].

Trying to promote stress tolerance in *Bmi1*+DR^IMM-REV^ cells, we evaluated some modifications in the culture conditions (Supplementary Materials; Section S2.7; Tables S3 and S4). As main criteria to define a positive effect of any novel culture condition, we tested its effect on *Bmi1* expression of treated *Bmi1*^+^DR^IMM^ after culture for 72 h (Figure S3A); unfortunately, none of the evaluated culture conditions provoked a relevant enhancement of *Bmi1* expression in the *Bmi1*+DR^IMM-REV^ population, which progressed similarly towards senescence (Figure S3B). Similar results were obtained when different inhibitors (see Supplementary Materials and Methods; Section S2.7; Table S5) of several pathways involved in Senescence-Associated Secretory Phenotype (SASP) were tested [38]. We observed no difference in terms of senescence (% of β-gal+ per field) in *Bmi1*^+^DR^IMM-REV^ cells transduced with Adeno-Cre (MOI200) and maintaining each condition during the GV selection (Figure S3C,D). Only treatment with rapamycin (mTOR inhibitor) allowed a statistically significant reduction in senescence cell (β-gal+) emergence (Figure S3C); however, simultaneous rapamycin, joined to the highest proliferative restriction, provoked a net reduction in cells per field (Figure S3D). On the other hand, Y27632 treatment (inhibitor of ROCK1) increased the number of cells per field (Figure S3D) but it did not counteract the evolution towards senescence, compared with control *Bmi1*^+^DR^IMM-REV^ cultures (Figure S3C). In conclusion, although some moderate improvements have been achieved in the conditions for managing *Bmi1*+DR^IMM-REV^ cultures, they do not allow working with them for further in vitro research.

### 2.4. Bmi1+DR^IMM^ Cells Show Comparable Oxidative Stress Sensitivity to Primary Bmi1+DR Cells

As described before, cardiac *Bmi1*+DR cells displayed a perivascular distribution, the low-ROS niche being generated by vasculature, proposed as the major regulator of *Bmi1*+DR population activity. In previous studies, pharmacological disruption of the endothelial barrier triggered a significant reduction in the percentage of *Bmi1*+DR cells [19], showing that integrity of the vascular barrier is relevant for the maintenance of *Bmi1*+ cells and pointing to a possible protective mechanism from cardiac endothelium. To achieve a deeper analysis of this, we decided to test the impact of the already-described Pq treatment in in vitro *Bmi1*+DR cell studies with endothelial cells.

**Figure 4:**
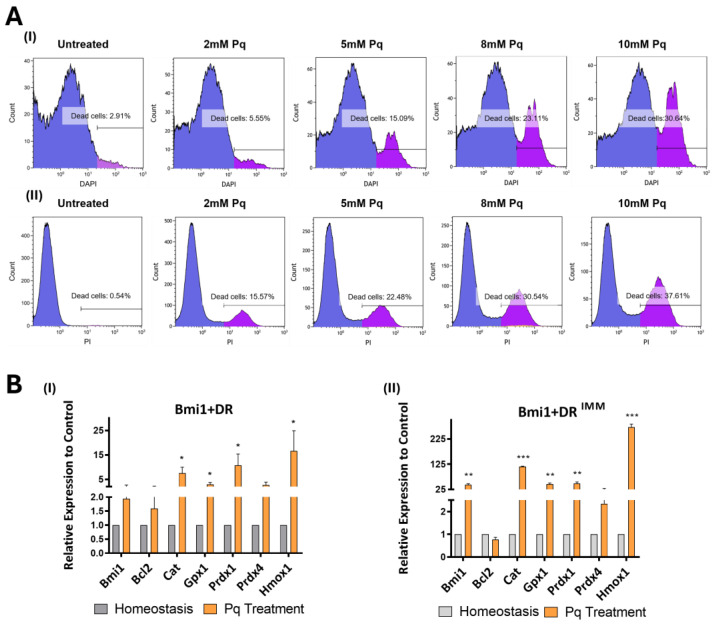

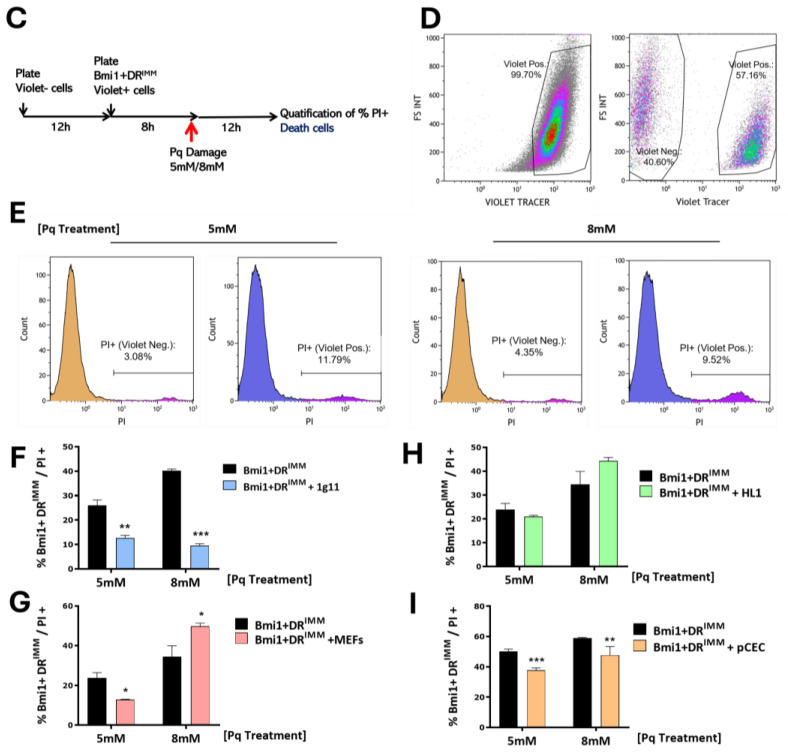
Co-culture with cardiac endothelium reduces the impact of oxidative stress damage in *Bmi1*+DR^IMM^ cells. (**A**) Cell death caused by Pq treatment at different concentrations in primary *Bmi1*+DR (DAPI+) and *Bmi1*+DR^IMM^ (PI+) cells analyzed by flow cytometry; cell damage labels compatible with the fluorescent proteins expressed by each line. (**B**) Evaluation by RT-qPCR of marker gene expression in response to oxidative damage induced by Pq treatment (5 mM; 12 h) on primary *Bmi1*+DR (I) and *Bmi1*+DRIMM (II) cells; expression represented as values relative to control untreated *Bmi1*+DR cells (homeostasis) (*n* = 3). (**C**) Timeline of the procedure followed to evaluate the severe oxidative damage response induced by in vitro Pq treatment on *Bmi1*+DR^IMM^ cells co-cultured with different cell types. (**D**) Cellular separation by flow cytometry of the co-cultured Violet+ cells (*Bmi1*+DR^IMM^ cells) and Violet- cells (other specific cell type). (**E**) Representative independent flow cytometry analysis of percentage of dead (PI+) Violet- cells and Violet+ cells (*Bmi1*+DR^IMM^ cells) under co-culture conditions and oxidative stress exposure (Pq treatment; 5 mM, 8 mM). Percentage of PI-labeled dead *Bmi1*+DR^IMM^ cells (% *Bmi1*+DR^IMM^/PI+) observed under oxidative damage conditions (5 mM or 8 mM Pq treatment; 12h) in the co-cultures carried out on (**F**) 1g11 cells; (**G**) MEFs; (**H**) HL-1 cell line; and (**I**) primary cardiac endothelial cells (pCECs) (*n* ≥ 3). Statistical analyses: * *p*-value < 0.05; ** *p*-value < 0.01; *** *p*-value < 0.001; Mann–Whitney U-Test.

Nevertheless, we first aimed to evaluate oxidative stress sensitivity in *Bmi1*+DR^IMM^ compared to *Bmi1*+DR cells, so we treated both *Bmi1*+DR^IMM^ and primary *Bmi1*+DR cells with increasing concentrations from 2 to 10 mM of Pq for 12 h (see Section 4.7) using untreated cells as controls. After these treatments, we labeled oxidative stress-associated death cells. In the case of *Bmi1*+DR^IMM^ cells, we stained dead cells with Propidium Iodure (PI+ dead cells); however, as primary *Bmi1*+DR cells were isolated from Bmi1-Tomato mice and *Tomato+* labelling could interfere with PI staining, we used DAPI as live/dead labelling (DAPI+ dead cells). Results (Figure 4A) demonstrated a similar Pq sensitivity (30.64 and 37.61% of dead cells in *Bmi1*+DR and *Bmi1*+DR^IMM^, respectively, using 10 mM Pq). Moreover, we checked the modulation of a panel of genes previously described as participating in the oxidative stress response to Pq damage in the mouse heart [39]. In *Bmi1*+DR vs. *Bmi1*+DR^IMM^ treated with 5 mM Pq for 12 h, we found also a similar response (Figure 4B); only *Bcl2* showed a differential behavior and a moderate exacerbated response in *Bmi1*+DR^IMM^ cells compared with *Cat*, *Gpx1*, *Prdx1,* and *Hmox1*. It is worth noting that *Bmi1* is similarly overexpressed (approx. 2-fold) upon Pq damage in both cell models (Figure 4B).

### 2.5. Co-Culture with Cardiac Endothelium Reduces the Impact of Oxidative Stress Damage in Bmi1+DR

Once we confirmed that *Bmi1*+DR^IMM^ population oxidative stress sensitivity and response are similar to primary *Bmi1*+DR cells, trying to go deeper into the endothelium niche regulatory role, as well as a plausible protection capacity against oxidative stress damage, we aimed to confirm this relationship in vitro and evaluate the specificity of the regulation. To test the putative effects of endothelium on *Bmi1*+DR viability, we cultured *Bmi1*+DR^IMM^ cells in acute oxidative stress damage conditions generated by Pq (5 or 8 mM, for 12 h) and in combination with different cell types as co-culture, including the established endothelial cell line 1g11 (see Section 4.8). The pipeline followed in the co-culture experiments is represented in Figure 4C. Due to the size of the reporter molecule, YFP has proven to be lost in dead *Bmi1*+DR^IMM^ cells (Figure S4A); thus, in order to differentiate both cell types in co-culture conditions and avoid eventual interference with the detection of apoptotic PI+ cells, *Bmi1*+DR^IMM^ cells were labeled in advance with Violet Cell Tracer (VCT) (Figure S4B).

Then, by flow cytometry, we could distinguish 1g11 (VCT-) and *Bmi1*+DR^IMM^ (VCT+) in co-culture and identify the dead cells (PI+) in each population (Figure 4D). Previously, we analyzed the damage profiles of both cell types co-cultured compared to individual cultures (mono-culture) under Pq treatment conditions (5 mM, 8 mM; 12 h). The 1g11 cells showed 4.32% of PI+ cells (quite resistant), while *Bmi1*+DR^IMM^ cells showed 30.67% PI+; however, when *Bmi1*+DR^IMM^ cells were co-cultured with 1g11 cells, being simultaneously subjected to the same oxidative damage condition (5 or 8 mM Pq), 1g11 cells did not modify their level of death (4.35%), but clearly, *Bmi1*+DR^IMM^ cells showed a significant reduction (9.52% in 8 mM PQ) within PI+ events (Figure 4E,F). We then evaluated the result of co-culturing *Bmi1*+DR^IMM^ in direct contact with the other two majoritarian cell types in the adult heart: the mouse cardiomyocytic-like cell line HL-1 (Figure 4G) and MEFs (fibroblasts) (Figure 4H). In clear contrast to the co-culture of *Bmi1*+DR^IMM^ cells with 1g11, HL-1 cells or MEFs did not substantially modify the level of oxidative damage (PI+ events) of *Bmi1*+DR^IMM^ cells (Figure 4G and H, respectively). Surprisingly, in MEF co-culture, the percentage of dead *Bmi1*+DR^IMM^ cells was only reduced when the lower Pq dose was used; the higher dose tested, however, promoted an increment in the *Bmi1*+DR^IMM^ PI+ cells (Figure 4H), so the protector effect observed in the endothelial co-culture was discarded. Moreover, the protector effect of endothelial 1g11 cells against oxidative damage on *Bmi1*+DR^IMM^ cells was demonstrated to be preferent; the co-culture of 1g11 with HL-1 cells showed non-significant modification of the level of PI+ cells in the HL-1 population at both Pq doses evaluated (5 and 8 mM) (Figure S4C). In addition, the reverse protector effect (from *Bmi1*+DR^IMM^ to the other cell types of partners in the co-cultures) was also evaluated, but no significant alterations were found (Figure S4D,F). Analysis of cell death in untreated cell mono-culture of Violet+ (*Bmi1*+DR^IMM^) and Violet- (1g11) confirmed the effect of Pq treatment (Figure S4G). Finally, to reinforce this hypothesis of the protector effect of endothelial cells under the conditions closest to in vivo conditions, we isolated primary cardiac endothelial cells (pCECs) from adult WT mouse hearts and repeated the same co-culture experiments with *Bmi1*+DR^IMM^ cells (see Section 4.4 and Section 4.8); results confirmed that when *Bmi1*+DRIMM cells were co-cultured with pCECs, being simultaneously subjected to the same oxidative damage condition (5 or 8 mM Pq), the percentage of dead *Bmi1*+DR^IMM^ cells showed a significant reduction in both conditions compared to individually cultured (mono-culture) *Bmi1*+DR^IMM^ cells (Figure 4I). These results strongly support the proposal for a *Bmi1*+DR endothelial niche (as the minimal exponent in an envisioned highly complex entity, by comparison with more developed niche models) capable of controlling their proliferative rate and protecting them from deleterious oxidative damage.

### 2.6. Regulation of Bmi1+DR Cardiac Progenitor Autophagy and Metabolic Status by Direct Contact with Cardiac Endothelial Cells

**Figure 5:**
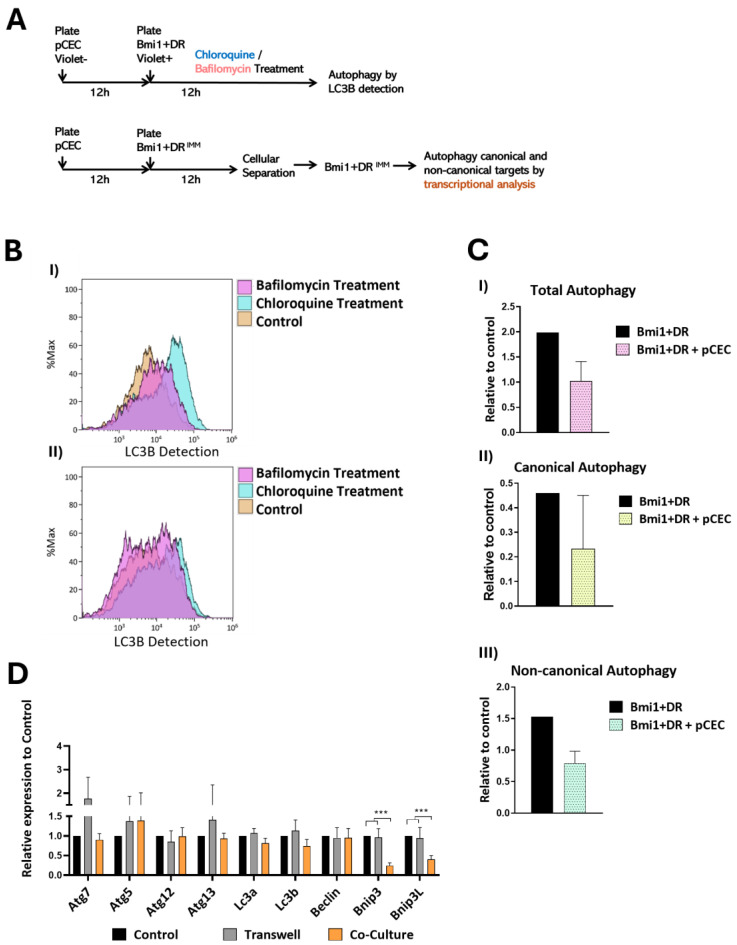
Direct contact with primary cardiac endothelial cells alters autophagic flux in *Bmi1*+DR cells. (**A**) Scheme and timeline of the procedure followed to evaluate the effect of the co-culture with primary cardiac endothelial cells (pCECs) on *Bmi1*+DR cell autophagy by LC3B detection and comparative transcriptional analysis. (**B**) Representative analysis of the L3CB detection by flow cytometry using LC3B antibody comparing control *Bmi1*+DR cells cultured independently (I) vs. *Bmi1*+DR cells co-cultured with pCEC in direct contact conditions (II) and (**C**) the corresponding quantitative analysis of the total (I), canonical (II), and non-canonical (III) autophagy flux (*n* = 3). (**D**) Expression by RT-qPCR in *Bmi1*+DR^IMM^ cells of genes involved in canonical and non-canonical autophagy represented as values relative to control *Bmi1*+DR^IMM^ cells cultured independently vs. *Bmi1*+DR^IMM^ cells co-cultured with pCEC separated by a transwell or in direct contact conditions. (*n* = 4) Statistical analyses: *** *p*-value < 0.001; one-way ANOVA Bonferroni post-test.

**Figure 6:**
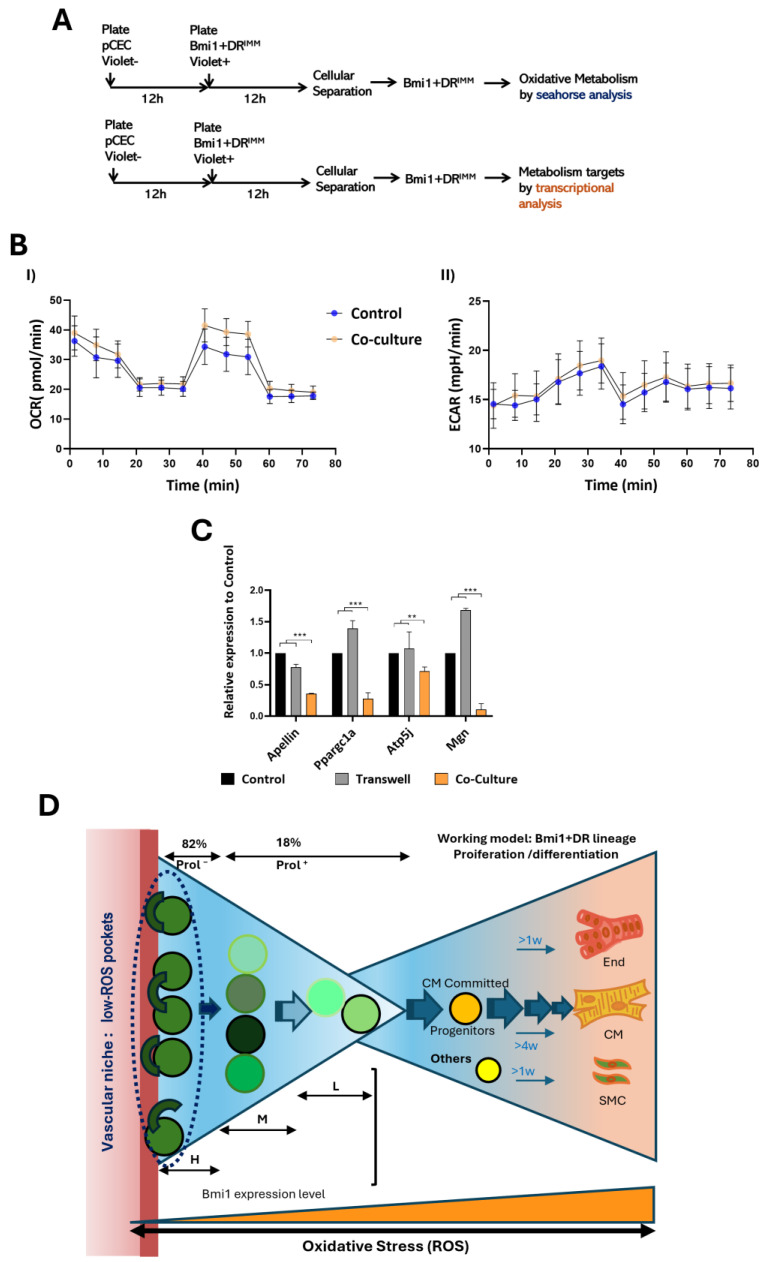
Direct contact with primary cardiac endothelial cells might reduce metabolic activity in *Bmi1*+DR^IMM^ cells. (**A**) Scheme and timeline of the procedure followed to evaluate the effect of the co-culture with primary cardiac endothelial cells (pCECs) on *Bmi1*+DR^IMM^ cells metabolic activity by Seahorse XF96 metabolic flux analyses and comparative transcriptional analysis. (**B**) Seahorse analysis profile of glycolysis and mitochondrial function by measuring (I) percentage of oxygen consumption rate (OCR) and (II) percentage of extracellular acidification rate (ECAR), both comparing *Bmi1*+DR^IMM^ cells cultured independently (control) and in direct contact with pCEC (co-culture) (*n* = 3). (**C**) Expression in *Bmi1*+DR^IMM^ cells of relevant genes involved in metabolism analyzed by RT-qPCR; expression was represented as values relative to control *Bmi1*+DR^IMM^ cells cultured independently vs. *Bmi1*+DR^IMM^ cells co-cultured with primary cardiac endothelial cells (pCEC) separated by a transwell or in direct contact conditions (*n* = 4). Statistical analyses: ** *p*-value < 0.01, *** *p*-value < 0.001; one-way ANOVA Bonferroni post-test. (**D**) Working model: The proposed vascular niche allows the protection of *Bmi1*+DR cells, maintaining them mostly in a non-proliferation state with a low rate of differentiation.

There is the notion that ASCs reside, especially the more preserved and primitive subpopulations, in hypoxic niches that help to maintain their immatureness and multipotency (reviewed by [40]). In general, self-renewal promotion seems to be associated with an enhanced autophagy and a preferred glycolic metabolism profile [41] that counteract oxidative damage-related OXPHOS overactivation (reviewed by [7]), promoting quiescence [42,43]. Similarly, *Bmi1*+DR cells, in the adult heart, are confined to discrete low-ROS domains associated with the vascular endothelium and demonstrate one of the lowest endogenous ROS levels within the organ [15,19]. Trying to understand the molecular mechanisms involved in this niche relationship with the cardiac endothelium, we evaluated the impact of direct or non-contact co-culture of *Bmi1*+DR^IMM^ cells with the closest population to their niche, primary cardiac endothelial cells, pCECs, and compared with individually cultured *Bmi1*+DR^IMM^ cells as control. Then, we analyzed the functional impact of these co-cultures on the control of cell autophagy flux and metabolic activity of *Bmi1*+DR^IMM^ cells, as well as the modulation in the expression of different genes putatively involved in those processes by RT-qPCR analysis (Figure 5A and Figure 6A).

We first evaluated the potential regulation of autophagy by monitoring the LC3B signal on *Bmi1*+DR cells in co-culture with pCECs (Figure 5B(I)), or individually cultured as control (Figure 5B(II)), using Chloroquine and Bafilomycin treatments to estimate total, canonical, and non-canonical autophagy (see Section 4.9). Figure 5C shows results obtained from the relative data of all co-culture data (*n* = 3), where we detected a decrease in total autophagy (Figure 5C(I)) and non-canonical autophagy (Figure 5C(III)) estimation, but not in canonical autophagy (Figure 5C(II)) with respect to the control. These results were further confirmed by the analysis of a panel of canonical and non-canonical autophagy-associated target genes, comparing their expression in individually cultured *Bmi1*+DR^IMM^ cells, non-contact (transwell), and direct contact co-culture with pCECs (Figure 5D). Results demonstrated that the co-culture did not significantly modify the level of expression of typical autophagy-associated genes (*Atg7*, *Atg5*, *Atg12*, *Atg133*, *Map1lc3a*, *Map1lc3b*, *Beclin*), but we observed a moderate reduction in *Lc3a* and *Lc3b* expression (confirming results in the previous functional assay) and a significant downregulation of *Bnip3* and *Bnip3L* gene expression (Figure 5D). Among other distinct roles for *Bnip3* and *Bnip3L*, genes related to mitochondria autophagy, they have been directly implicated in autophagosome formation and recognition and cardiac myocyte autophagy. Therefore, we concluded that, unexpectedly, direct contact of pCECs with *Bmi1*+DR^IMM^ cells alters autophagic flux in *Bmi1*+DR cells, but not through the promotion of canonical autophagy.

In parallel to the previous study, and concerning the plausible role of niche in metabolic regulation of ASCs, we checked the impact of pCECs co-cultured with *Bmi1*+DR^IMM^ cells on the metabolic activity of the progenitor population (Figure 6A). After further purification of *Bmi1*+DR^IMM^ cells, we evaluated the two main metabolic pathways, mitochondrial respiration and glycolysis, using the Agilent Seahorse XF platform (see Section 4.10). Results indicated that, upon co-culture, *Bmi1*+DR cells showed minimal changes in higher Oxygen Consumption Rate (OCRI), particularly at the basal and maximal respiration rate, which could be indicative of a moderate OXPHOS increase (non-mitochondrial respiration seemed negligible) (Figure 6B(I)), while the Extracellular Acidification Rate (ECAR) showed no apparent changes in glycolytic flux (Figure 6B(II)). Then, we tried to confirm these conclusions, analyzing the modulation of a panel of selected genes critically involved in metabolism regulation and comparing their expression in individually cultured *Bmi1*+DR^IMM^ cells, non-contact (transwell), and direct contact co-culture with pCECs (Figure 6A,C). We found a significative downregulation of *Apelin* (Figure 6C), which promotes the import and consumption of glucose and fatty acids [44]. A low level of *Apelin* expression, therefore, could be associated with a slowing of *Bmi1*+DR cell metabolism upon co-culture with primary endothelial cells. Furthermore, we found a significant parallel reduction in *Ppargc1a* expression (Figure 6C), a main regulator of oxidative metabolism; in agreement with this, *Atp5j*, a direct target of *Ppargc1a* [44], is also significatively inhibited (Figure 6C). On the contrary, *Ndufb5* (NADH-Ubiquinone Oxidoreductase SGDH Subunit; Complex I SGDH Subunit) was unaffected by the co-culture with pCECs (Figure 6C). All regulated genes seem to be mainly dependent on *Bmi1*+DR direct contact with endothelial cells. In conclusion, although the impact of pCEC co-culture with *Bmi1*+DR cells did not show important differences in main metabolic pathways, some critical genes involved in OXPHOS regulation were clearly downregulated; this could favor a moderately lower rate of mitochondrial respiration, but not a clear concomitant enhancement of glycolytic flux. Globally, this situation may be associated with the inherently low ROS levels and the concomitant quiescence-prone and undifferentiated state of *Bmi1*+DR cells, although other relevant regulatory pathways could be involved (Figure 6D).

## 3. Discussion

The niche relationship among different ASC populations and the endothelium has been widely defined in several organs/compartments, for example, in neural stem cells (NSCs) [45,46,47], bone marrow (BM)-derived cells [48,49], and skeletal muscle stem cells (MuSCs) [50,51]. In our previous studies, we confirmed the spatial relationship observed between *Bmi1*+DR cells and the cardiac endothelium [19]. Then, in an attempt to try to understand the role of the *Bmi1*+DR population in cardiac turnover and response to damage, we consider a key point to unravel their potential niche-like relationship with cardiac endothelium.

This niche relationship is envisioned as critical for the maintenance and fate of *Bmi1*+DR cells. Previously, it was confirmed that after AMI (5–10 d), *Bmi1*+DR cells are not apparently damaged; they were even proliferatively activated [18], moderately increasing their numbers, and as the main consequence, long after AMI (4 months), a substantial increase in the number of mature cells is demonstrated (Figure S1B). Here, we confirmed that a similar behavior after Pq treatment (single dose, 5 d post-Tx) is inducible in a Bmi1-Tomato mouse model (Figure 1A(II)); monitoring of heart cryosections of the *Tomato+* (*Bmi1*+) signal, and cell numbers, relative to the total (DAPI+) cells, also showed a moderate expansion (Figure 1E). Similarly, it is established in mammalian arteries that smooth muscle cells (SMCs), a highly resting population in human arteries, only contribute efficiently to repair when drastic damage is inflicted [44].

Cardiac endothelium, as the proposed niche for the *Bmi1*+DR population, might control its cell distribution, differentiation potential, and proliferative status. A previous study showed that *Bmi1*+DR cells display a perivascular gradient-like cell distribution in the adult mouse heart, with only a small percentage of these cells (≈10% of total *Bmi1*+ cells) being in a proliferative state [19]. Moreover, Herrero at al. showed how oxidative damage conditions modified *Bmi1* activity in vivo by derepressing canonical target genes in favor of their antioxidant and anticlastogenic functions to trigger ROS-associated differentiation of this cardiac progenitor population, pointing out that the differentiation potential of *Bmi1*+DR cells is clearly mainly controlled by oxidative stress [17]. In addition, we found that, specifically, only perivascular areas with very low ROS levels coincided with the localization of the majority of *Bmi1*+DR cells in vivo, which is very similar to those described in other ASC compartments [29], but this specific distribution was distorted when general low-ROS conditions were applied by genetically decreasing ROS levels in Bmi1-Tomato mice using G6PDTg, which confirmed the importance of perivascular ROS levels [19]. On the contrary, here we showed that when general high-ROS conditions were applied with Pq treatment, *Bmi1*+DR cell preference to be close to the endothelium increases, showing a tighter relationship with the endothelium in response to damage (Figure 1F). All of these data reinforce the vascular niche hypothesis for *Bmi1*+DR cells, with oxidative stress levels being a major regulator (Figure 1G).

In order to achieve a deeper analysis of this niche-like relationship of *Bmi1*+DR cells with cardiac endothelium, and to unravel the regulatory mechanisms, we decided to carry out an analysis in cultures of *Bmi1*+DR and endothelial cells, as conducted previously [19]. However, due to the scarcity of *Bmi1*+DR cells, we faced significant experimental limitations, as we were restricted to working with primary cultures, in addition to the great number of mouse models needed to try to understand the physiology of this progenitor population. Then, aiming to facilitate the dissection of the plausible mechanisms that could play a relevant role involving endothelial cells and *Bmi1*+DR cells, as well as to reduce the needs for animal models, we tried to develop a reversible immortalization procedure for *Bmi1*+DR cells.

After revision and evaluation of the previous literature, we decided to use the expression of the SV40-T that was successfully used with similar cell types [31,32]. Other strategies involved the overexpression of *Bmi1* and *TerT*, which were both discarded because of the central role of *Bmi1* expression in *Bmi1*+DR cells. In addition, it was demonstrated that in primary cardiomyocytes, SV40-T is a superior immortalization agent when compared with *Bmi1* and *TerT* [36]. We aimed to immortalize the *Bmi1*+DR population, isolated from the inducible Bmi1-YFP mouse model (*Bmi1*+/+) (Figure 1A(I)) [15,52]. This population was successfully immortalized, generating the *Bmi1*+DR^IMM^ pool, which showed a clear exponential and maintained (more than 40 passages) proliferation (Figure 2C). The *Bmi1*+DR^IMM^ population was characterized, demonstrating a similar expression of relevant cell surface makers, global expression profiles, and biological responses to several signaling molecules, previously tested to define *Bmi1*+DR cells [15,16,17,18,19] (Figure 2F–H). As previously described, *Bmi1*+DR cells were initially considered as a mixed population of mutually exclusive PDGFRα+ and CD31+ cells. Although flow cytometry of *Bmi1*+DR^IMM^ showed negative results for CD31 (Figure 2F) expression, we considered that a strong post-transcriptional effect could be involved or the PDFGRα+ subpopulation could have a higher proliferating rate [18]. Then, *Bmi1*+DR^IMM^ should represent the *Bmi1*+DR/PDGFRα+ cells.

Nonetheless, our first attempt was to develop a reversible immortalization procedure where, upon the transient expression of Cre-Recombinase, the LoxP flanking sequences would recombine and delete the immortalization cassette; furthermore, this construct also expresses TK to eliminate cells that would not be properly engineered. Unfortunately, although reversibility of SV40-T immortalization has been described [23,53,54], the procedure on *Bmi1*+DR^IMM^ provoked a sudden proliferation block and the induction of senescence (Figure 3). This phenotype has been also found in other cell types, such as human olfactory ensheathing glia [35]. This result could not be rescued by modifications of the culture medium nor by the addition of senescence inhibitors (Figure S3). In conclusion, although we could not fully use the *Bmi1*+DR^IMM^ platform as planned, the *Bmi1*+DR^IMM^ population was confirmed as an incredibly useful tool to study these cardiac progenitor cells, postulating this immortalization procedure to be applied for the study of other complex adult progenitor populations. Altogether, the *Bmi1*+DR^IMM^ population seems to be quite a reasonable model for the study of *Bmi1*+DR cells endothelial niche.

The niche concept was first proposed by R. Schofield for HSCs and referred to the surrounding supporting cells and the soluble factors that influence HSC behavior [55]. Currently, the niche is generally considered the real functional unit in most ASC compartments, being critically responsible for tissue or organ homeostasis, damage response (regeneration), and repair [6,7,56]. Among all ASC models, HSCs [57,58], NSCs [45,59], and MuSCs [50,51] are, perhaps, the best-known compartments. Many data postulate that MuSCs [60] and *Bmi1*+DR cells [19] showed a similar interaction with their corresponding microvasculature, mainly controlling them by redox regulation [13,17,18,19,61]. One of the main goals of the potential cardiac vascular niche regulation is to reduce the impact of the progressive oxidative stress in the progenitor populations during adulthood, aging, or cardiac damage conditions. However, to our knowledge, no study has addressed the plausible direct impact of experimental oxidative stress levels (acute or chronic) on the biology of MuSCs and their niche, as well as the main cell–cell interactions contributing to the preservation of MuSCs and counteracting oxidative stress; research has been concentrated in ischemia- and reperfusion-derived damage aging [62], as well as in other experimental dedicated models. Then, in the cardiac tissue, we asked whether vasculature could protect *Bmi1*+DR cells against substantial ROS levels.

In this regard, we first analyzed *Bmi1*+DR^IMM^ cells response to oxidative damage by in vitro Pq treatment and demonstrated quite a similar response to the non-immortalized population (Figure 4A,B), as well as to the analysis of the whole heart [39]. Next, we modelled in vitro the “minimal” cardiac endothelial niche, co-culturing *Bmi1*+DR^IMM^ cells (or, when indicated, primary *Bmi1*+DR cells) with the 1g11 endothelial cell line or primary cardiac endothelial cells (pCECs), and evaluated the functional consequences of short acute oxidative stress conditions (Pq, 12 h) compared with individually cultured *Bmi1*+DR cells (mono-culture) and other majoritarian cell types in the cardiac tissue. Results clearly indicate that co-culture of *Bmi1*+DR^IMM^ with the 1g11 endothelial cell line and pCECs, but not with embryonic fibroblasts (MEFs) or the mouse cardiomyocyte-like cell line HL-1, promotes a protective effect for resistance to medium–high Pq concentrations (5 or 8 mM) (Figure 4F–I). The effect was dose-dependent and specific, as HL-1 cells were not protected by endothelial co-culture (Figure S4C), and the effect was unidirectional, as *Bmi1*+DR^IMM^ cells did not exert any protective effect on the other cell populations (Figure S4D–F). Therefore, it can be concluded that endothelial cells seem to play a notable role in preserving *Bmi1*+DR maintenance from oxidative stress. This consideration must be related to the high in vivo resistance of *Bmi1*+DR cells to several forms of damage, including AMI, irradiation, mitomycin [17], and Pq. This protective role of niche endothelial cells in stem cell populations was first described in the lymphohematopoietic system after radiation [63] and in cardiac resident populations. Moreover, it was previously described that the cardiac Sca1+ CD31- subpopulation protects cardiomyocytes against different forms of damage, included AMI, being mediated by MCP-1 [64] and potentiated by miR-133a [65].

On the other hand, a central and solid result concerned the substantial reduction in *Bmi1*+DR intracellular ROS by direct co-culture with pCECs [19], so we evaluated the regulation of the main mechanisms that could contribute, including autophagy and metabolism shift, as revealed for skeletal muscle [66]. Autophagy is key in preventing stresses as one of the major quality control guardians in the cell; the autophagy pathways acquire physiological relevance even under basal, non-stressful conditions, being especially relevant for the maintenance of stem cell self-renewal potential [67,68], cellular differentiation, and plasticity [69]. Tissues that are mainly composed of post-mitotic/quiescent cells exhibit higher sensitivity to loss of autophagy competence. For example, in the skeletal muscle, MuSCs display a continuous basal level of autophagy critical for their stemness and maintenance capacity, showing that physiological decline in autophagy in old satellite cells or its genetic impairment in young cells results in toxic cellular waste accumulation and progression towards senescence [70]. In this context, we aimed to evaluate whether primary endothelial cells (pCECs) could modulate autophagy in *Bmi1*+DR cells, after direct contact co-culture of these cells. Results indicated that, unexpectedly, autophagy was not enhanced but, on the contrary, total and non-canonical autophagy flux was moderately reduced by co-culture with pCECs. This result was further confirmed by RT-qPCR analysis of the panel of genes relevant for autophagy; none of them were enhanced but, on the contrary, *Bnip3L and Bnip3*, critically involved in mitophagy (selective autophagic degradation of mitochondria) [44,71], appeared quite significantly reduced only in direct contact co-cultures (65 and 80% reduction, respectively). According to this, in the context of aging, basal autophagy was found to be reduced in a subset of younger HSCs compared to their older counterparts [72]. In this way, while mitophagy might be critical for clearing metabolically active mitochondria to maintain quiescence in some stem cell populations [72], the role of mitochondria is key in NSCs, as mitochondria regulate self-renewal by maintaining low levels of ROS [73]. Therefore, although we did not find direct evidence that pCECs, in the basal stage, promote autophagy in *Bmi1*+DR^IMM^ cells by cell–cell contact, it is possible that the reduction in non-canonical autophagy and mitophagy-associated gene expression might be associated with the regulation of ROS levels by the endothelial niche. Moreover, some critical signals (molecules or other cell types) could be lost from this minimal vascular niche.

Nonetheless, another critical mechanism involved in intracellular ROS control among ASC populations is the regulation of their metabolic activity. Indeed, their relationship with the vascular niches has been also related with metabolic control. For example, HSCs in homeostasis reside close to their vascular niche, which promotes an enhanced glycolytic state, reducing ROS production [74]. Equally, the opposite effect has been observed in studies performed in zebrafish, with an enhanced oxidative metabolism in endothelial cells as an initiator of the revascularization process after cardiac damage [75]. Then, we decided to study the effects on *Bmi1*-DR^IMM^ cell metabolism of pCEC direct co-culture. The analysis of the two main metabolic pathways did not render important differences; the co-culture with pCECs compared to individually cultured *Bmi1*+DR cells rendered a moderately higher oxygen consumption rate index (OCRI, proportional to mitochondrial respiration), particularly for the maximal respiratory rate, but a similar basal respiration and no differences in extracellular acidification rate (ECAR, proportional to glycolysis) were found. However, in a second analysis by RT-qPCR, we observed that co-culture with pCECs reduced *Apelin* expression in *Bmi1*+DR^IMM^ cells, a critical metabolic mediator that activates import and consumption of glucose and fatty acids [76], *Ppargc1a*, a master regulator of oxidative metabolism and some targets, and *Atp5j* [77]. These modifications of expression profile would suggest that co-culture with pCECs promotes a metabolic slow-down, as in other stem cell compartments [41]. Accordingly, quiescent hematopoietic stem cells exhibit low oxidative phosphorylation levels, switching to a high-oxidative-phosphorylation metabolic state only after their activation. Globally, further studies are required to determine if autophagy, or even mitophagy, and metabolic glycolytic state play a role in modulating the switch in ROS level dynamics between quiescent undifferentiated and differentiated *Bmi1*+DR cells by their endothelial niche.

While our current study demonstrates that the *Bmi1*+DR^IMM^ pool not only maintains long-term cell proliferation capacity but also retains their native *Bmi1*+DR cell counter-part characteristics, some differences and limitations should be pointed out. In relation to the immortalization process, the reversibility of SV40-T immortalization on *Bmi1*+DR^IMM^ cells was unsuccessfully achieved due to the fulminant senescent phenotype provoked; this anticipates limitations of their clean usage for certain applications. Obviously, we were interested in a well-controlled immortalization for expansion, followed by dis-immortalization prior to functional evaluations. The global phenotype seems to be related to the selection of the immortalizing function, because a previous publication using SV40-T found a similar picture, demonstrating that cell proliferation rate significantly decreased in selected clones of cardiac progenitor cells upon SV40-T removal [53]. In spite of that, all of the discussed evidence indicates that the *Bmi1*+DR^IMM^ pool reasonably resembles the main characteristics of primary *Bmi1*+DR cells, including some biological and oxidative responses [19], but reflecting also clear differences (Figure 2F–H); all data suggest that *Bmi1*+DR^IMM^ cells might better represent a *Bmi1*+DR/PDGFRα+ subpopulation. Overall, the *Bmi1*+DR^IMM^ pool was proven to be a useful tool, allowing us to recreate the minimal vascular niche using co-cultures and continue its definition. Further work with the *Bmi1*+DR^IMM^ pool has also demonstrated that they can be used for colony forming assays with quite similar results to primary *Bmi1*+DR cells, and even in in vitro forced differentiation assays. We are convinced that further work with *Bmi1*+DR^IMM^ cells will help to expand complexity in the cardiac minimal vascular niche similarly to the composition and regulation of the skeletal muscle niche, as the closest reference.

## 4. Materials and Methods

### 4.1. Transgenic Mice and Tamoxifen Administration

Transgenic mice used in this study, *Bmi1^CreERT/+^*-*Rosa26^YFP/+^, Bmi1^CreERT/+^*-*Rosa26^TdTomato/+^* and *Bmi1^GFP/+^* (all from The Jackson Laboratory), were maintained on the C57BL/6 background, as previously required [15,16,17,18,19]. All animal strains used were adult mice (8–12 weeks old); as previously indicated in previous studies and detailed in Supplementary Materials S2.1, they are used with the corresponding administrative and ethical authorizations.

For Tamoxifen (Tx) administration, Tx (Sigma-Aldrich Inc., St. Louis, MI, USA, T5648) was dissolved in corn oil (Sigma-Aldrich Inc., St. Louis, MI, USA, C8267) and intraperitoneally (i.p.) injected (225 μg/g body weight) in *Bmi1^CreERT/+^*-*Rosa26^YFP/+^* or *Bmi1^CreERT/+^*-*Rosa26^TdTomato/+^* animals every 24 h for 3 days. The animals were used, fundamentally, for the different experiments 5 days after finishing the induction.

### 4.2. Immunofluorescence of Cardiac Tissue and Image Analysis

The extraction of cardiac tissue was performed 5 days after the last dose of Tx, or 48 h after paraquat (Pq) administration. Once the animals were anesthetized and sacrificed, the heart was perfused with 1X PBS through the hepatic vein to clean the remains of blood from the ventricular and atrial cavities. After that, the heart was kept rotating for 12 h at 4 °C in a solution of 4% paraformaldehyde (PFA; TED PELLA, Redding, CA, USA, 18505) for fixation. Afterwards, the heart was kept in increasing sucrose solutions in a gradient of 15 to 30% concentration for dehydration. This allows final inclusion in OCT (Sakura Finetek Spain S.L., Barcelona, Spain, 25608-930) of the heart. Using a microtome, histological sections of 6–8 µm thickness were obtained from the ventricular zone of each heart for subsequent analysis by immunofluorescence.

The histological heart sections were treated for 1 h at room temperature (RT) with 1X PBS + 3% bovine serum albumin (BSA; Sigma-Aldrich Inc., St. Louis, MI, USA, A7906). The permeabilization of the membrane was performed with the detergent Triton X-100 dissolved at 0.5% in 1X PBS, incubating the sections at RT for 20 min. After several washes with 1X PBS + 1% BSA, histological sections were incubated with blocking solution 1X PBS + 5% BSA for 2 h at RT. After washing again with 1X PBS + 1% BSA, sections were incubated overnight at 4 °C with the corresponding primary antibodies, Rabbit anti-αSMA (Abcam, Cambridge, England, ab5664) and Rat anti-Sca1 (RyD systems, Minneapolis, MN, USA, MAB1226), diluted to 1:50 and 1:100, respectively, in a solution of 1X PBS + 1% BSA + 0.1% Triton X-100). The next day, sections were washed with 1X PBS + 1% BSA and subsequently incubated for 1 h at RT with the corresponding secondary antibodies (anti-Rabbit 647 nm and anti-Rat 488 nm (Jackson, Bar Harbor, ME, USA, 111-176-104) prepared at 1:500 in the same solution of the primary antibody. After several washes with 1X PBS + 1% BSA, sections were incubated with DAPI (Sigma-Aldrich Inc., St. Louis, MI, USA, D9542) diluted in 1X PBS at a concentration of 1:500 for 20 min at RT. Finally, sections were set up with ProLong Antifade Mountant (Invitrogen, Madrid, Spain, P36930). The resulting immunofluorescence of the cardiac tissue was analyzed by imaging with the Leica SP5 Microfluor microscope and their subsequent processing and analysis with the Image J program version FIJI (National Institute of Health, Belthesda, MD, USA).

### 4.3. Isolation and Culture of Adult Mouse Non-Myocyte Bmi1+DR Cells

Primary non-myocyte cells and cardiomyocytes were obtained by the Langendorff method using retrograde perfusion through the aorta. The heart was removed rapidly and retrograde-perfused under constant pressure (60 mmHg; 37 °C, 8 min) in Ca^2+^-free buffer (113 mM NaCl, 4.7 mM KCl, 1.2 mM MgSO_4_, 5.5 mM glucose, 0.6 mM KH_2_PO_4_, 0.6 mM Na_2_HPO_4_, 12 mM NaHCO_3_, 10 mM KHCO_3_, 10 mM Hepes, 10 mM 2,3- butanedione monoxime, and 30 mM taurine). Digestion was initiated by adding a mixture of recombinant enzymes (0.2 mg/mL Liberase Blendzyme (Roche, Madrid, Spain, 05401127001), 0.14 mg/mL trypsin (ThermoFisher, Waltham, MA, USA, 15090046), and 12.5 μM CaCl_2_) to the perfusion solution. When the heart became swollen (10 min), it was removed and gently teased into small pieces with fine forceps in the same enzyme solution. Heart tissue was further dissociated mechanically using 2, 1.5, and 1 mm diameter pipettes until all large heart tissue pieces were dispersed. The digestion buffer was neutralized with stopping buffer (10% fetal bovine serum (FBS; Capricorn Scientific, Ebsdorfergrund, Germany, FBS-12A) and 12.5 μM CaCl_2_). Cardiomyocytes were pelleted by gravity in a two-phase decantation process (45 and 30 min, respectively), and the supernatant was used as a source of non-myocyte cardiac cells [14].

Primary *Bmi1+*DR cells were isolated from *Bmi1^CreERT/+^*-*Rosa26^YFP/+^* (*Bmi1*+DR YFP+ cells), *Bmi1^CreERT/+^*-*Rosa26^Tomato/+^* (*Bmi1*+DR *Tomato+* cells), and *Bmi1^GFP/+^* (*Bmi1*+DR GFP+ cells) mice by cell sorting with the corresponding reporter after Langendorff digestion and expanded in Iscove’s modified Dulbecco’s medium (IMDM; ThermoFisher, Waltham, MA, USA,12440-053) supplemented with 10% FBS, 100 IU/mL penicillin (Invitrogen, Madrid, Spain), 100 mg/mL streptomycin (Invitrogen, Madrid, Spain), 10^3^ units ESGRO-LIF (Millipore, Burlington, MA, USA, ESG1107), 20 ng/mL FGF (Fibroblast Growth Factor; Peprotech,100-18B), 10 ng/mL EGF (epidermal growth factor; Peprotech, AF-100-15), and 100 μg/mL Normocin (InvivoGen, San Diego, CA, USA, ant-nr-1). *Bmi1*+DR cells were cultured under hypoxic conditions (37 °C, 3% O_2_, 5% CO_2_) and culture plates previously treated with 0.1% gelatin.

### 4.4. Isolation and Culture of Adult Mouse Primary Cardiac Endothelial Cells

For the isolation of primary cardiac endothelial cells (pCECs), wild-type (WT) mice that did not include any genetic modification were used. After euthanizing the animals, the hearts were perfused with 1X PBS through the vena cava to eliminate circulating hematopoietic cells in the chambers of the heart that could interfere with the subsequent extraction process. The heart was removed and was mechanically disintegrated with the help of a scalpel. Once the heart was disintegrated into the smallest fragments possible, we proceeded with enzymatic digestion using DMEM medium supplemented with Collagenase (Sigma-Aldrich Inc., St. Louis, MI, USA, C5138) and Dispase II (Hoffmann-La Roche, Basel, Switzerland, 04 942 076 001), both at a concentration of 1 mg/mL, at 37 °C for 45 min under stirring. Homogenization of the resulting solution was performed by passing it through a sterile 18 G needle and a 70 µm sterile filter (Sigma-Aldrich Inc., St. Louis, MI, USA, 352350) to eliminate possible large fragments not digested correctly. Isolation medium (DMEM supplemented with 20% FBS, 100 U/mL penicillin, and100 µg/mL streptomycin) was added and the resulting cell suspension was centrifuged at 400 g for 5 min. After washing with 1X PBS + 0.5% BSA, cells were centrifuged at 300× *g* for 10 min.

pCECs were isolated from the obtained pellet by magnetic separation using the MACS Neonatal Cardiac Endothelial Cell kit Isolation Kit (MACS Miltenyi Biotec, Bergisch Gladbach, Germany, 130-104-183) and expanded in VascuLife VEGF Endothelial Medium Complete Kit (Lifeline Cell Technology, San Diego, CA, USA, LL-0003). pCECs were cultured under normoxic conditions (21% O_2_, 5% CO_2_, 37 °C) in plates previously treated with 1% gelatin and supplemented with 100 μg/mL fibronectin (Sigma-Aldrich Inc., St. Louis, MI, USA, F1141); cells were used for the experiments at passage ≤4–5.

### 4.5. Culture Conditions for Other Cell Lines

The detailed culture conditions for the different cell lines used in the study are detailed in Supplementary Materials S2.2.

### 4.6. Immortalization/Dis-Immortalization of Bmi1+DR Cells

#### 4.6.1. Immortalization, Transduction of *Bmi1*+DR Cells with the Lentiviral LoxP-SV40 T-Large–TK-LoxP Vector, and Further Expansion/Confirmations

To obtain the immortalized pool of primary *Bmi1*+DR, cells were isolated and sorted from *Bmi1^CreERT/+^*-*Rosa26^YFP/+^* (*Bmi1*+DR YFP+ cells) or *Bmi1*^GFP/+^ (*Bmi1*+DR GFP+ cells) mice and transduced with the immortalization lentiviral vector (pLOX-Ttag-iresTK; Addgene, Watertown, MA, USA, 12246); the composition of the vector is depicted in Figure 2A. The pLOX-Ttag-iresTK vector was produced by the viral vector production unit at the National Center for Cardiovascular Research (CNIC) (Madrid, Spain), and it is a 3rd-generation lentiviral vector, in terms of biosafety. Several batches were produced in HEK 293T, pseudotyped for VS.V-G; the co-transfection was carried out using pLOX-Ttag-iresTK + vector VS.V-G (pMD2.G; Addgene, Watertown, MA, USA, 12259) + vector Pax2 (psPax2; Addgene, Watertown, MA, USA, 12260), using lipofectamin 3000 (Invitrogen, Madrid, Spain, L3000). The titer of the different batches was estimated by RT-qPCR using standard curves.

Primary *Bmi1*+DR cells were transduced with the lentiviral vector supernatant at the indicated MOIs 1–10; cells were seeded in 6-well plates using an 80% confluency (7000 cells/cm^2^). Lentiviral transduction was carried out in OPTIMEM (ThermoFisher, Waltham, MA, USA, 31985-070) supplemented with polybren (Sigma-Aldrich Inc., St. Louis, MI, USA, TR-1003) at 8 μM. Cells were maintained in the conditions previously described for *Bmi1*+DR culture for 24 h, then cells were washed, and culture medium was refreshed and maintained for an additional 24 h. Finally, transduced cells were maintained in standard culture conditions, with subcultures carried out every three days until confluency was reached. The scheme of the followed procedure can be found in Figure 2B.

For monitoring insert status by PCR, total DNA was extracted from the cells using the NucleoSpin Tissue extraction kit (Macherey-Nagel, Düren, Germany, 740952). The PCR reaction was carried out in an Applied Biosystems Veriti 96 thermoblock well (Applied Biosystems, Waltham, MA, USA) according to the following program: 10 min at 95 °C, 40 cycles of 15 s at 95 °C, 1 min at 60 °C, 30 s at 72 °C, and 7 min at 72 °C. Amplification was performed using the same primers as in the detection by RT-qPCR. For analysis, visualization of the amplificated section was performed by loading the PCR result into a 1.5% agarose gel (Condalab, Madrid, Spain, 8010.22) stained with Ethidium Bromide (Sigma-Aldrich Inc., St. Louis, MI, USA, E1610). Bands were confirmed using markers of suitable size (1 Kb DNA Plus Ladder; ThermoFisher, Waltham, MA, USA, 10787018).

#### 4.6.2. Analysis of the Expression of Membrane Markers by Flow Cytometry

As part of the validation of the *Bmi1*+DR^IMM^ immortalized population, we obtained an expression profile by flow cytometry for some of the markers of the membrane that characterize the *Bmi1*+DR population. *Bmi1*+DR^IMM^ cells were amplified in culture until there were 10^6^ cells for each marker to analyze. The cells were trypsinized and washed twice with 1X PBS. Possible non-specific targets were blocked by 1 h incubation of cells in suspension at RT with 1X PBS + 5% BSA. After the blocking, the cell suspensions were incubated with each of the primary antibodies against the membrane markers to analyze. The marking was applied for 1 h at RT under gentle rotation to avoid precipitation of the cells. Subsequently, two washes were carried out with 1X PBS + 5% BSA and followed by labeling with the secondary antibodies under the same conditions as the primary labeling. The antibodies conjugated with fluorochromes that were used only required a marking step. The antibodies used and their concentrations of use are listed in Table S2. Finally, fluorescently labeled cells were detected using Gallios Flow Cytometer (Beckmann Coulter, Madrid, Spain), and results were analyzed using Kaluza Analysis software version c1.2.1. (Beckmann Coulter, Madrid, Spain).

#### 4.6.3. Evaluation of *Bmi1*+DR^IMM^ Response Assays to Recombinant Proteins

With the aim of determining whether *Bmi1*+DR^IMM^ cells keep some of the functional characteristics of primary *Bmi1*+DR cells, *Bmi1*+DR^IMM^ cells were seeded in culture plates covered with 0.1% gelatin and cultured in medium supplemented with the indicated factors and conditions described in Supplementary Materials S2.3. Total RNA was extracted and expression levels of *Bmi1* were analyzed by RT-qPCR using the primers shown in Table S1*;* modulation of *Bmi1* expression was compared with that of primary challenged *Bmi1*+DR^IMM^ cells.

#### 4.6.4. Dis-Immortalization: Reversal of SV40-T Immortalization by Transient Expression of Cre Recombinase

The adenoviral vector (Adeno-Cre) (SignaGen Laboratories, Frederick, MD, USA, SL100707) was prepared by the viral vector service at the CNIC (Madrid). For the vector preparation, HEK293T cells were also used, and the crude vector preparations were purified with the Adeno X-Purification Kit (Taxara, San José, CA, USA, 632249) kit, and the viral titer was established using the kit Adeno X-^TM^-rapid titer Kit (Taxara, San José, CA, USA, 632250). *Bmi1*+DR^IMM^ cells were seeded in 6-well plates to a confluency of 80% (7000 cells/cm^2^). Cells were maintained for 24 h in transduction medium, and then *Bmi1*+DR^IMM^ cells were transduced with Adeno-Cre using several MOIs (1, 2, and 5 × 10^2^ infective particles for each cell); cells were incubated for an additional 72 h, in conditions equivalent to the transduction with lentiviral vector. After a step of washing the culture, medium was exchanged and the culture was kept for an additional 24 h to allow them to recover, and then the expression of the immortalizing function and selection marker (RT-qPCR and Western Blot) was monitored. The feasibility of de-immortalization was previously demonstrated [34]. Effective Cre-dependent activity should delete the floxed cassette; in addition, treatment with ganciclovir (InvivoGen, San Diego, CA, USA, ant-nr-1). SUD-GCV) allowed us to eliminate those cells that did not delete the immortalization cassette. *Bmi1*+DR^IMM^ cells were seeded at 7000 cells/cm^2^ and, after 24 h, the negative selection with Ganciclovir (GCV, 1 μM) was added and maintained for 7 days. Associated with the expression of SV40, the cassette also expresses Timidin Kinase (TK), which metabolizes GCV, inducing cell death; this will eliminate cells that did not eliminate the immortalization cassette. The process was monitored for the reversion of the immortalization and posterior selection. Those *Bmi1*+DR^IMM^ cells that were manipulated with the Adeno-cre and survived to GCV selection were denoted *Bmi1*+DR^IMM-REV^ cells. The scheme of the followed procedure can be found in Figure 3A.

#### 4.6.5. Evaluation of Proliferative Status of *Bmi1*+DR Cells

##### Evaluation of Cell Proliferative Status through Population Doubling Rate

Population doubling rate was quantified in parallel in all treatments of immortalization for *Bmi1*+DR cells compared. For this, all crops were reseeded at the same time, and in each passage, the same number of cells was seeded. The passages were performed every 3 days, and 7000 cells/cm^2^ were reseeded. In the case that it was not reached, this minimum number of cells between one passage and the next, the total of the cells present in each condition was considered for the calculation of the doubling rate in the next pass. The cells were kept in culture for as long as possible, given that the control cells and those not effectively immortalized entered a stationary phase in which there was minimal or no cell proliferation. To calculate the parameter of the population doubling rate, we use the following formula:

Population doubling rate = [log (N° Counted cells) − log (N° Seeded cells)]/log2

##### Evaluation of Cell Proliferative Status through EdU Incorporation

To evaluate the proliferative status using the labeling EdU (5-ethynyl-2′-deoxyuridine), we incubated the cells for 12 h in standard culture medium supplemented with 10 µM EdU. Labelling for proliferative cells was performed with Click-iT EdU Alexa Fluor 647 nm Flow Cytometry Kit (Invitrogen, Madrid, Spain, C10424) following the manufacturer’s instructions. EdU^+^ cells were detected fluorescently within each population using the Gallios Flow cytometer (Beckmann Coulter Madrid, Spain) and results were analyzed using Kaluza Analysis software version c1.2.1. (Beckmann Coulter, Madrid, Spain).

#### 4.6.6. Analysis of Cellular Senescence by β–Galactosidase Staining

Staining was performed against cells in a state of senescence based on the activity of β-galactosidase. The commercial Senescence β–Galactosidase kit (Cells Signaling Technology, Danvers, MA, USA, 98605) was used following the protocol indicated by the manufacturer. To determine the number of cells senescent per field, images were taken with the Olympus IX70 microscope (Olympus, Tokyo, Japan) and labeled cells were quantified Image J program version FIJI (National Institute of Health, Belthesda, MD, USA)

In addition, we evaluated several strategies to improve the survival and proliferation of *Bmi1*+DR^IMM-REV^ dis-immortalization (see Tables S3–S5).

### 4.7. Paraquat Treatments

Treatments with paraquat (N,N′-dimethyl-4,4′-bipyridinium dichloride) (Pq; Sigma-Aldrich Inc., St. Louis, MI, USA, 36541) were carried both in vivo or in vitro. For in vivo treatment, *Bmi1^CreERT/+^Rosa26^Tomato/+^* animals, 5 d post-Tx induction, were injected (i.p.) with a single dose of Pq (20 mg/kg body weight, diluted in 1X PBS), as previously described [19]. Pq-treated animals were sacrificed, and hearts were analyzed 48 h later. For in vitro treatment, cell populations (primary or immortalized) were treated with Pq after washing once with 1X PBS and the culture medium was replaced with the corresponding culture medium without any supplement, and under culture conditions of the target cell type whose effect was analyzed, including 5 or 8 mM Pq. Treatment was maintained for 12 h and then analyzed from the different aspects. When indicated, the target population was previously labelled with Violet Tracer.

### 4.8. Co-Culture Experiments

To evaluate the effects of cell–cell contact between *Bmi1*+DR^IMM^ cells and other cell types present in the heart through co-culture, we treated the culture surface with gelatin 0.1 or 1% and the corresponding supplements depending on the highest concentration required by the cells used in the co-culture. Subsequently, we seeded the first cell type at 25,000 cells/cm^2^ (HL-1, MEFs, 1 g 11 or pCECs) in its culture medium. After 8 h, we verified that these cells had adhered correctly, removed their medium culture, washed with 1X PBS, and seeded the *Bmi1*+DR^IMM^ cells on top at the same density. All analyses were conducted after 12 h of co-culture in the corresponding medium and culture conditions of the target cell type whose effect was analyzed. When indicated, co-cultures were compared with non-contact cultures using transwells (Transwell Permeable Supports 0.4 μm Polycarbonate Membrane; Sigma-Aldrich Inc., St. Louis, MI, USA, 3412) to avoid cell contact.

On the one hand, we analyzed the effect of co-cultures on the survival of the different cell types studied against exposure to severe oxidative damage (Pq treatment, Section 4.7). Because the low size of the fluorescence marker YFP in *Bmi1*+DR^IMM^ cells was significantly diffused after Pq treatment, confusing results (see Figure S4), we used cells previously labelled with the CellTrace™ Violet reagent (ThermoFisher, Waltham, MA, USA, C34557) at a concentration of 5 μM in a ratio of 1 million cells/mL 1X PBS, incubating for 20 min in darkness at 37 °C. After the administration of Pq, co-culture was maintained with the co-culture for an additional 12 h, before analysis. Dead cells were analyzed for propidium iodide staining (PI; Abcam) or DAPI staining. After the Pq treatment, dead cells labeled with propidium iodide (PI; Abcam) or DAPI (Beckman Coulter) within each population (Violet-/Violet+) were detected using the Gallios Flow Cytometer (Beckmann Coulter) and results were analyzed using Kaluza Analysis software version c1.2.1. (Beckmann Coulter, Madrid, Spain).

On the other hand, we analyzed gene expression in co-cultures of *Bmi1*+DR^IMM^ cells with pCECs. First, we separated the different cell types present in each co-culture. To accomplish this, we trypsinized the cells and separated them by FACS. This technique allowed the separation of fluorescently labeled *Bmi1*+DR^IMM^ (YFP+) cells from the negative without any type of labeling. Once the different fractions were separated, we extracted total RNA and analyzed the variations in expression with respect to the *Bmi1*+DR^IMM^ mono-culture as control by RT-qPCR.

### 4.9. Autophagy Evaluation by LC3B Detection, Difference between Total and Canonical Autophagy

Direct contact co-culture of pCECs with *Bmi1*+DR cells was performed as described (see Section 4.10). LC3B detection was carried out using the Guava Autophagy LC3-antibody-based assay Kit (Luminex, Austin, TX, USA, FCCH100171), following the instructions described by the manufacturer. Due to the detection of LC3B antibody (488 nm), *Bmi1*+DR^IMM^ cells (YFP+) were not suitable; in this case, we performed co-culture of pCECs with primary *Bmi1*+DR cells (labelled prior with Violet tracer). Co-culture and mono-culture, as control, were maintained for 12 h under *Bmi1*+DR culture conditions using *Bmi1*+DR culture medium supplemented with bafilomycin (10 μM), chloroquine (40 μM), and without supplement as control. Treatments with bafilomycin and chloroquine, as previously described [78], allowed us to discriminate between canonical and non-canonical autophagy. Anti-LC3B 488 nm detection (% Max intensity) was performed using Gallios Flow Cytometer (Beckmann Coulter, Madrid, Spain) and results were analyzed using Kaluza Analysis software version c1.2.1. (Beckmann Coulter, Madrid, Spain). Total autophagy was calculated as the ratio of Anti-LC3B detection of [(Chloroquine treated cells−Untreated cells)/Untreated cells], Canonical autophagy as the ration of Anti-LC3B detection of [(Bafilomycin treated cells−Untreated cells)/Untreated cells], and finally, non-canonical autophagy as the difference between total and canonical autophagy.

### 4.10. Metabolism Activity by Seahorse Analysis

Direct contact co-culture of pCECs with *Bmi1*+DR^IMM^ cells was performed as described (see Section 4.8). Co-culture was maintained for 12 h, then pCECs were separated from the co-culture by magnetic separation using the MACS Neonatal Cardiac Endothelial Cell Isolation Kit. *Bmi1*+DR^IMM^ cells were seeded in specific cell culture microplates and metabolic activity was carried out by Agilent Seahorse XF96 kit (Agilent technologies, Madrid, Spain, V3-PS TC-Treated, 101085-004). Results were analyzed in XF96 Analyzer obtaining the percentage of oxygen consumption rate (OCR) and the percentage of extracellular acidification rate (ECAR), as previously described [79].

### 4.11. Statistical Analysis

Statistical analyses were carried out with GraphPad Prism 7.0 software. For the study of data composed of a number of experimental samples greater than 15 (*n* > 15), the distribution was analyzed using the Shapiro–Wilk test, considering a normal distribution when *p* ≥ 0.05. In the analysis of experiments composed of two conditions, the Mann–Whitney U-Test was used. For experiments in which multiple conditions were analyzed, the Kruskal–Wallis test followed by Dunn’s post-test was used in the case of samples with parametric non-distribution or the one-way ANOVA test followed by Bonferroni post-test in the case of comparisons with parametric distribution. Significant differences were considered in the experiments that had a *p*-value less than 0.05 (* *p* < 0.05, ** *p* < 0.01, *** *p* < 0.001).

## 5. Conclusions and Future Perspectives

We have obtained and characterized a heterogeneous pool of immortalized *Bmi1*+DR^IMM^ cells, using SV40-T, from an adult cardiac population enriched in multipotent progenitors. Phenotypical and genomic characterization of this immortalized model resembles main features of the original *Bmi1*+DR population. Then, with the logical limitations, *Bmi1*+DR^IMM^ cells have proven to be a useful tool for dissecting the mechanisms involved in their regulation, both in homeostasis and in response to damage, although only in certain contexts. In conjunction with primary cardiac endothelial cells and the 1 g 11 endothelial cell line, we have modeled the minimal vascular niche using co-cultures. This strategy has allowed us to demonstrate a preferent protection of *Bmi1*+DR by endothelial cells against oxidative damage, contributing also with modest metabolic regulation and scarce involvement of autophagy.

In conclusion, as in other ASC models, and particularly in MuSCs, a perivascular niche for *Bmi1*+DR is envisioned. A better comprehension of the regulation of the cardiac niche(s) would be key for resolving uncertainties about the involvement of cardiac progenitor/stem cells on heart homeostasis and damage repair, and to demonstrate whether the low margin of heart turnover is relevant for healthy aging or in some pathological scenarios.

## Data Availability

The data presented in this study are available on request from the corresponding author.

## Supplementary Materials

The following are available online at https://www.mdpi.com/article/10.3390/ijms25168815/s1. Refs. [80,81,82,83] are cited in the Supplementary Materials.
