# Supplementary material for: Development of a Bmi1+ Cardiac Mouse Progenitor Immortalized Model to Unravel the Relationship with Its Protective Vascular Endothelial Niche
Source: Int J Mol Sci. 2024 Aug 13;25(16):8815. doi: 10.3390/ijms25168815 (PMC11354400; doi:10.3390/ijms25168815)
Supplement: Supplementary file 1 [file ijms-25-08815-s001.zip › ijms-3113310-supplementary.pdf]

S1. Supplementary Materials and Methods

S1.1. Main Supplementary Materials. Tamoxifen (Tx; Sigma, T5648); corn oil (Sigma, C8267); N,N′-dimethyl-4,4′-bipyridinium dichloride, Paraquat; Pq; Sigma 36541); 5-ethynyl-2′-deoxyuridine (EdU; Sigma, T511285); ganciclovir (1 µM; GCV, InvivoGen; Ref. SUD-GCV); iodide staining (PI; Abcam Ref ab14083) or DAPI (4',6-diamidino-2-phenylindole; Beckman Coulter, Barcekine, Soain); liberase Blendzyme (Roche, 05401127001); fetal bovine serum (FBS; Capricorn, FBS-12A; pLOX-Ttag-iresTK lentiviral vector, (from Addgene,1224, lipofectamin 3000; Invitrogen, L3000); adenoviral vector, Adeno-Cre, (SignaGen Laboratories; Ref#: SL100707); Transwell Permeable Supports, 0.4 µm Polycarbonate Membrane; from Corning Costar (Ref. 3412); the CellTrace™ Violet kit (ThermoFisher Sciencitific; Ref C34557);

S2. Supplementary Methods

S2.1. Transgenic Mice Experimental Design and Authorization

Experiments were carried out in male and female mice as recommended by the US National Institutes of Health since preliminary analysis revealed no differences between males and females [80]. 8-20 weeks-old mice were used as adult mice unless otherwise indicated in the text. The animals were stabled in the CNB-CSIC facilities with water and food ad-libitum. All procedures performed to the animals were approved by the Ethics Committee of the CNB-CSIC and the Division of Animal Protection of the Community of Madrid (PA 56/11, PROEX 048/16 and PROEX 250.5/20). Each of the procedures carried out were proposed based on the EU Directive 2010/63EU and Recommendation 2007/526/EC for the protection of animals used for experimentation and other scientific purposes, included in Royal Decree 1201/2005.

S2.2. Cell Culture Conditions for Cell Lines

Immortalized murine embryonic fibroblasts (MEFs), kindly provided by Prof. Carlos Martínez-A, (CNB-CSIC), were cultured in standard conditions (5% de CO2, 21% de O2, 37oC) using DMEM supplemented with FBS (10%), penicillin (100 U/ml), streptomycin (100 µg/ml) and L-Glutamine (2 mM).

The 1g11 murine pulmonary endothelial-like cell [81] was a kind gift of Dr. S Mañes (CNB-CSIC). 1g11 were cultured in (37 °C, 21% O_2, 5% CO_2) in culture plates previously treated with 0.1% gelatin, diluted in PBS. Culture medium was DMEM supplemmented with FBS (20%), penicillin (100 U/ml), streptomycin (100 µg/ml), 2mM L-Glutamine (Biowest; Ref. X0550), 0.1 mg/mL de ECGS (Endothelial Cell Growth Supplement, Sigma; Ref. E2759) and heparin (Sigma; Ref. H3393). Prior to be used in any experimental setting ECGS y heparin supplements were retired from the culture medium.

HL-1 cells are murine cardiomyocyte-like cells [82], provided by Dr. S Mañes (CNB-CSIC). They were grown in standard conditions (37 °C, 21% O_2, 5% CO_2) in culture plates previously treated with 0.1% gelatin, diluted in PBS, and supplemented with fibronectin (1 mg/ml). Cell growth

medium was Claycomb (Sigma, Ref. 51800C), supplemented with FBS (20%), penicillin (100 U/ml), streptomycin (100 µg/ml) and 2mM L-Glutamine.

S2.3. Recombinant Protein Assays

For EphrinB2 or EphB4 stimulation, clustered EphrinB2-Fc or EphB4-Fc (both from R&D Systems, 496-EB and 446-B4; 5 µg/ml) were absorbed on 0.1% gelatin-coated cell culture plates (2 h, 37 °C). Clustering was achieved using anti-human IgG, Fcγ fragment specific antibody (Jackson ImmunoResearch, 109-005-098) at a 2:1 M ratio, as described [83]. After 12 h, cells were detached from dishes and GFP fluorescence was measured by FACS. For the Fc protein binding assay, Bmi1^{+}DR-cells were detached from dishes, washed twice (5% FBS/PBS) and incubated with 2 µg/ml Fc-fusion proteins (1 h, 4 °C). After washing, cells were recovered and Bmi1 expresssion modulation was evaluated by RT-qPCR. For VEGFA stimulation, VEGFA (R&D Systems, 493-MV; 10 ng/ml) was added to Bmi1^{+} cardiac DR-cell cultures,12 h prior to analysis.

S2.4. Evaluation of Apoptosis

When indicated the impact of primary endothelial cells (pCEC) in the oxidative stress inflicted by Pq (0-8 mM) was evaluated in cardiac Bmi1+DR-cells; because the low size of the fluorescence marker, it was significantly diffused after treatment, confusing results (see Fig S4). Therefore, we used Bmi1+DR-cells previously labelled with the CellTrace™ Violet kit (ThermoFisher Sciencitific; Ref C34557) and analyzed for propidium iodide staining (PI; 3 mM/mL) (Abcam) or DAPI (Beckman Coulter) staining, by FACS. When possible, single cell analysis for the evaluation of apoptosis was carried out after DAPI staining (10 mM/mL).

S2.5. Evaluation of Senescence

For evaluation of cell senescence the Senescence β–Galactosidase Staining Kit (Cells Signaling Technology, Ref. 98605) was used. We followed the instruction by the manufacturer. For the quantification of senescence (SEN+) per field, images were taken with Olympus IX70 (Olympus) microscope and SEN+ per field were quantified with the ImageJ software (National Institutes of Health, EEUU).

S2.6. Western Blotting

For western blotting, cells were lysed (45 min, 4 °C) in radioimmunoprecipitation assay buffer (RIPA; Sigma-Aldrich, R0278), with addition of complete, EDTA-free Protease Inhibitor Cocktail (Roche, 04-693-132-001). Proteins were quantified using a Multiskan GO Spectrophotometer (Thermo Scientific). Lysates were size-fractionated by SDS-polyacrylamide gel electrophoresis, transferred to Hybond ECL nitrocellulose membranes (ThermoFisher, IB401002), probed with indicated antibodies (anti-SV40T and anti-tubuline; Table S2) and analyzed by enhanced chemiluminescence (GE Healthcare, RPN2209).

S2.7. Evaluation of Different Conditions to Improve the Promotion of Senescence after Dis-immortalization of Bmi1-DR^{IMM}

Aiming to improve the viability of the cultures of the Bmi1-CPC-YFP$^{Inm\text{-}Rev}$ model after dis-immortalization procedure we evaluated several factors and inhibitors that have been described to have activity on progenitors and stem cells. The we analyzed the effect of the described (Table S3)

agent and the composition of the culture medium that contain them (Table S4); we aimed to improve both survival and proliferation capacity of las Bmi1-CPC-YFP$^{Inm\text{-}Rev}$ model after dis-immortalization. Cells, with their corresponding controls were cultured in the indicated culture medium and compared with the standard conditions for Bmi1-CPC, for the subsidiary senescence state.

Additionally, several inhibitors of the Senescence Associated Secretory Phenotype (SASP); all they are indicated, as well the selected concentration, in Table S5. After dis-immortalization of Bmi1-CPC-YFP$^{Inm\text{-}Rev}$, treated with the Adeno-vector, they were re-seeded for the negative selection with GCV, during the evaluation of each inhibitor.

Supplementary Figures

A

Gene Name	Function	Expression Ratio Bmi1+Sca1+/Bmi1-Sca1+
Bmi1	Stem cell marker	*2-fold* ↑
Sca1	Stem cell marker	*1,5-fold* ↑
Actn2	*Interaction with angiogenina*	11-fold ↑
Tnnt2	*Muscle contraction*	10-fold ↑
Cd31	Angiogenesis	3.5-fold ↑
SCL (Tal1)	Endothelial and progenitor cells marker	3.2-fold ↑
VE-cadherin	Major endothelial adhesion molecule	3-fold ↑
Tie1	Control angiogenesis	2.5-fold ↑
Mcam (CD146)	Angiogenesis	3.5-fold ↑
vWF	Endothelial marker	2.5-fold ↑
Endoglin (CD105)	Endothelial marker	2-fold ↑
Vegfa	Angiogenesis	0.7-fold ↓
Vegfr1 (FLT1)	Angiogenesis	4.3-fold ↑

B

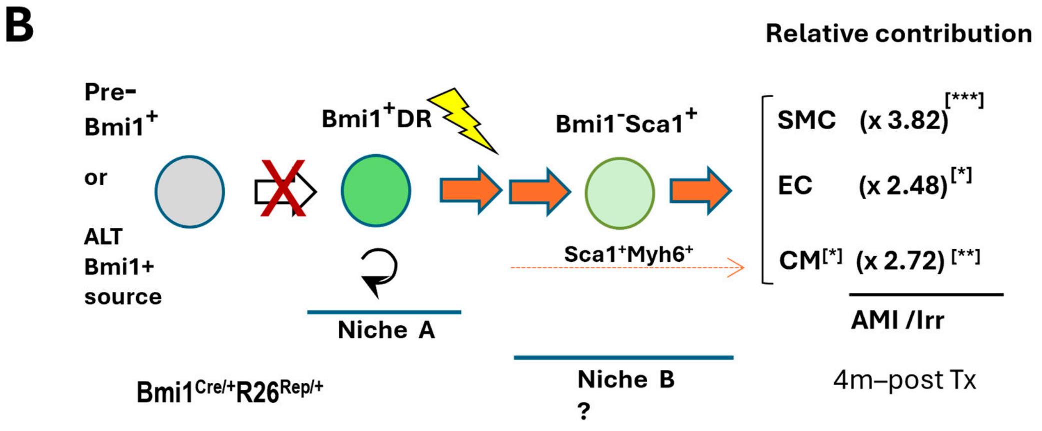

Figure S1. *Bmi1+DR presents a differentiation bias toward endothelial cells lineage.* (**A**) Endothelial-related genes upregulated in the Bmi1+DR population (Bmi1+ Sca1+) compared to its closest population in cardiac tissue (Bmi1-

Sca1+). (**B**) In response and upon damage (yellow ray indicated AMI) Bmi1+DR cells show a clear contribution to cardiac mature cells after AMI (4-months), including endothelial lineage.

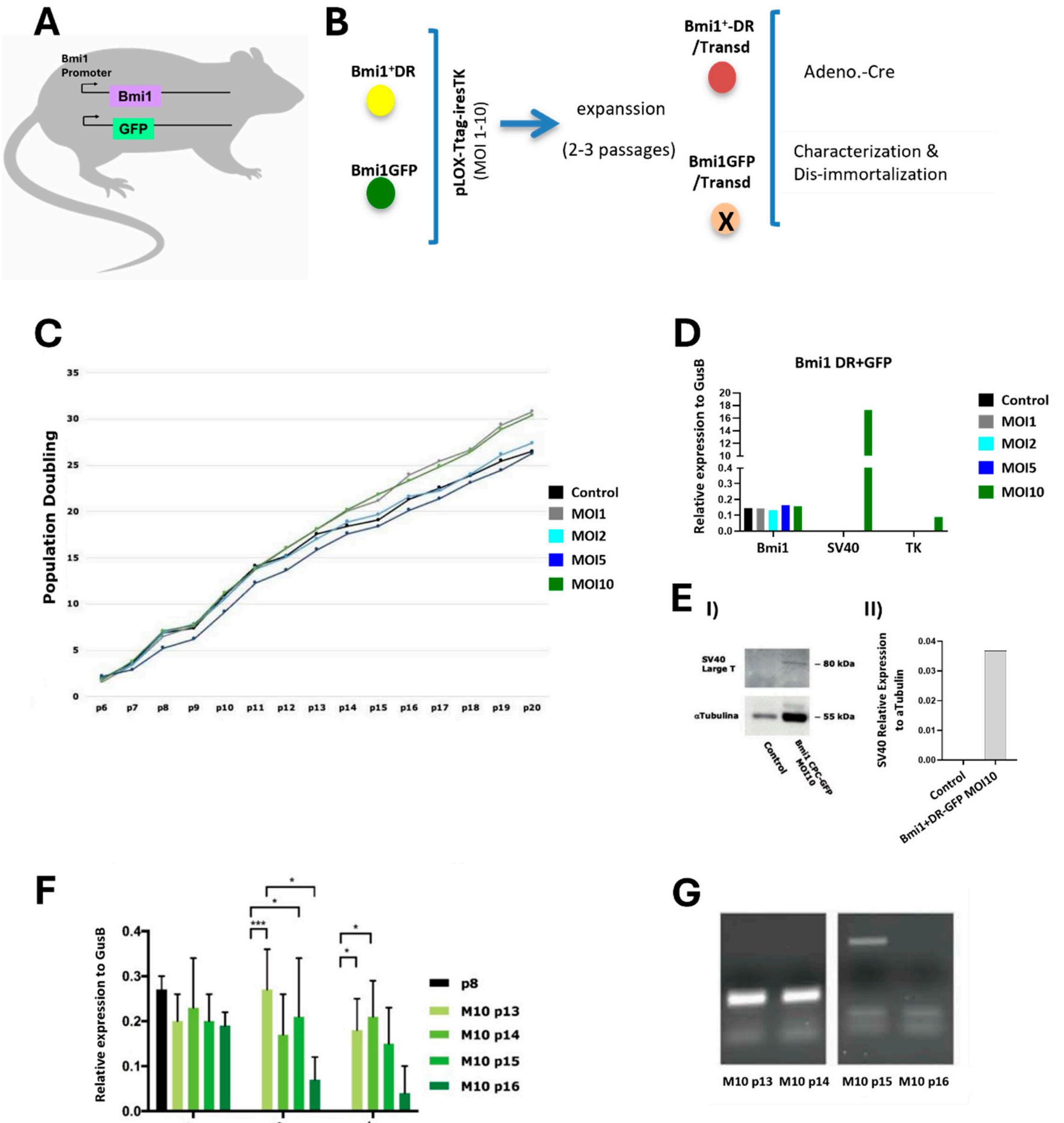

Figure S2. *Generation and characterization of a conditionally-immortalized Bmi1+GFP population.* (**A**) Animal model Bmi1$^{GFP/+}$. (**B**) Scheme of the procedure followed for the generation of the immortalized cell line through transduction of the T-SV40/TK lentiviral vector using parallelly primary Bmi1+DR cells from Bmi1$^{CreERT/+}$ Rosa26$^{YFP/+}$ mice or from Bmi1$^{GFP/+}$ mice [19, 33] (**C**) Representation of the progression of the cumulative population doubling rate (Y axis) after successive passes (p; X axis) of primary Bmi1+DR cells treated with the different indicated MOIs concentration. (**D**) Comparative RT-qPCR analysis of the expression of T-SV40 and TK relative to GusB in primary Bmi1+GFP cells treated with the different MOIs evaluated. (**E**) Representative Western Blot of the expression of the T-SV40 protein in Bmi1+DR cells transduced with MOI10 (I) and corresponding analysis of the expression levels of the T-SV40 protein relative to αTubulin as control (II). (**F**) Comparative RT-qPCR expression of SV40 and TK, relative to endogenous gene GusB, in immortalized Bmi1+GFP cells after successive passages (n=3). (**G**) Representative image of the genomic DNA fragment amplified for the T-SV40 gene in immortalized Bmi1+GFP cells from passage 13, 14, 15 and 16 (non-detectable). Statistical analyses: *p<0.05; ***p<0.001; Two-Way ANOVA Bonferroni post-Test.

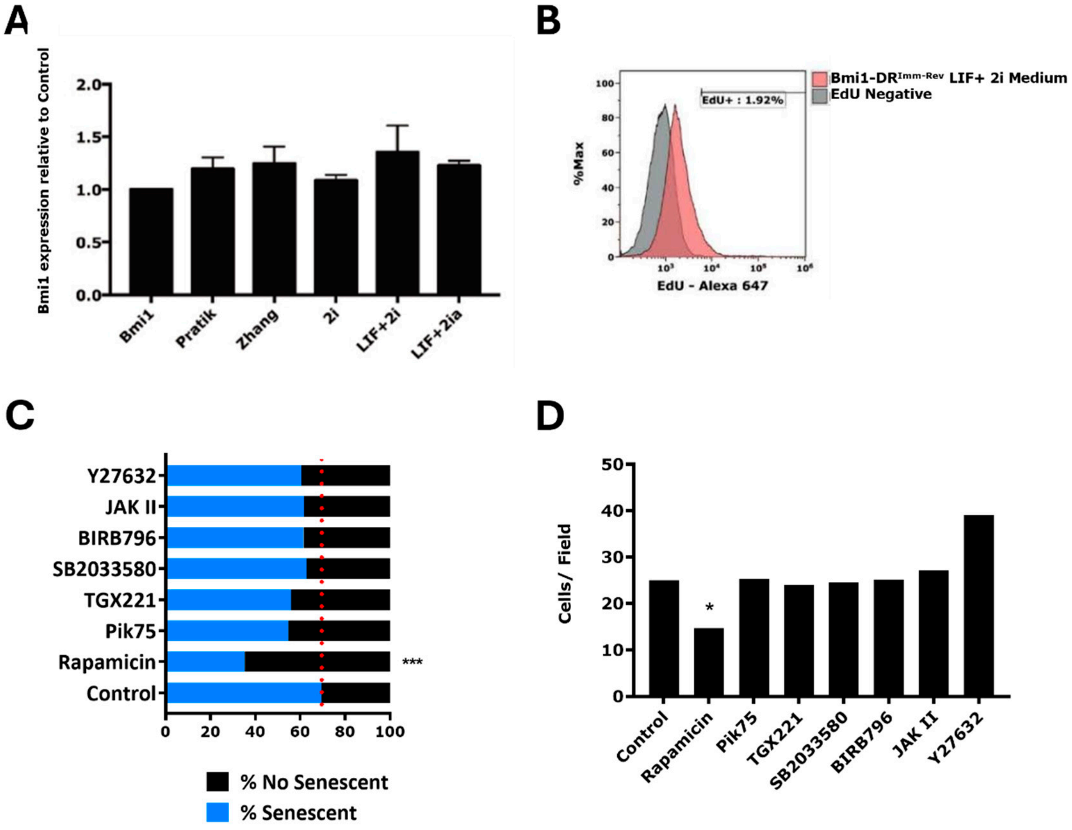

Figure S3. *The use of alternative culture media or inhibitors of signaling pathways involved in SASP do not represent a significant improvement for the culture of Bmi1+DR^{IMM} or Bmi1+DR$^{IMM-REV}$*. (**A**) Comparative RT-qPCR for the expression of Bmi1 in Bmi1+DR^{IMM} cells maintained under standard conditions after culture in the different media tested comparing to classical Bmi1+DR medium as Control (n=3). (**B**) Histogram indicative of proliferative Bmi1+DR^{IMM} EdU^{+} (Alexa647) cells after maintenance for 72h with LIF+2i medium. (**C**) Representation of the percentage of senescent cells (β-galactosidase staining) observed per field and (**D**) the corresponding quantification of the total number observed per field in the images analyzed in cultures of Bmi1+DR$^{IMM-REV}$ cells treated during the de-immortalization process with each of the SASP inhibitors; considering Bmi1+DR$^{IMM-REV}$ cells de-immortalized without no additional senescence inhibitor treatment as Control (n=3). Statistical analyses: *p<0.05, ***p<0.001; One-Way ANOVA Bonferroni post-Test.

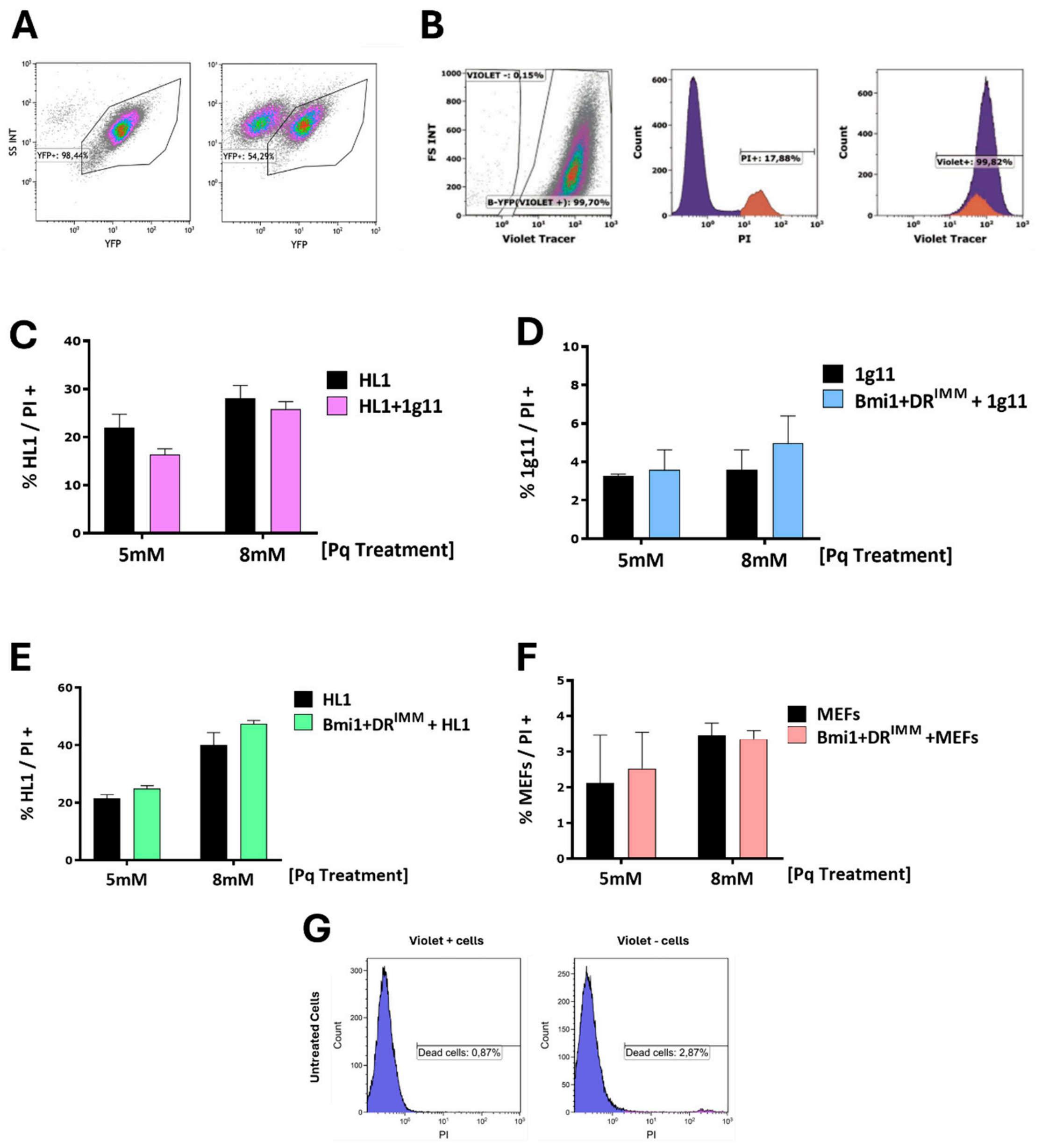

Figure S4. *The protective effect of the endothelial cells on Bmi1+DR^{IMM} cells is specific to the interaction between both cell types but Bmi1+DR^{IMM} cells do not present a protective effect on the different co-cultured cell types.* (**A**) YFP reporter is not compatible with the quantification of cell death but (**B**) Violet Tracer labeling is efficient and compatible with the quantification of cell death by PI after induction of oxidative damage by Pq treatment (5mM; 12h). (**C**) Quantification of the percentage of HL-1 PI-labeled dead cells (% HL-1/PI+) observed after induction of oxidative damage for 12h with 5mM or 8mM Pq treatment in the co-cultures carried out with 1g11(n=3). Quantification of the percentage of PI-labeled dead cells (% cell type/PI+) observed after induction of oxidative damage for 12h with 5mM or 8mM Pq treatment in the co-cultures carried out on (**D**) 1g11 cells, (**E**) HL-1 cells and (**F**) MEFs with Bmi1+DR^{IMM} cells (n=3). **(G)** Cell death analysis by flow cytometry of untreated Bmi1+DR^{IMM} cells (Violet+) and 1g11 cells (Violet-).

Complete WB for Fig 2E - S2E

Exposition 1min, Anti-SV40 dil 1:100

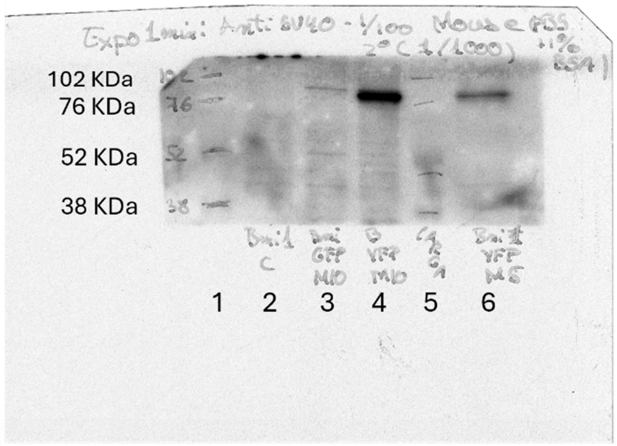

1-Ladder
2-Control
3-Bmi1 DR-GFP MOI10
4-Bmi1 DR-YFP MOI10
5-Ladder
6-Bmi1 DR-YFP MOI5

Exposition 1min, Anti-αTubuline dil 1:1000

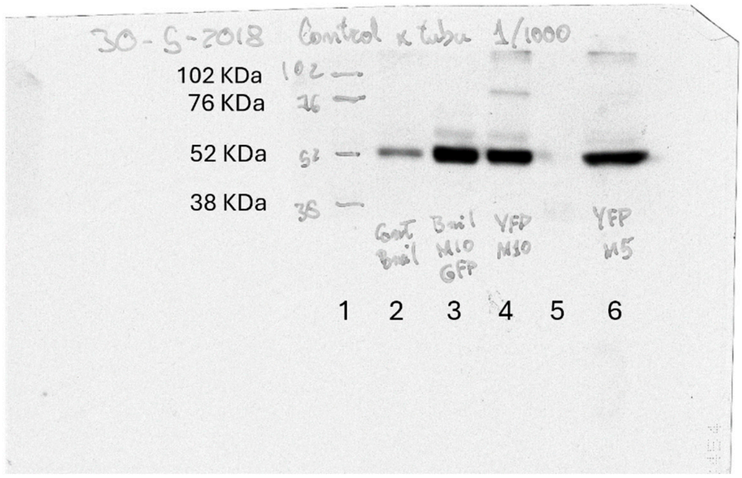

1-Ladder
2-Control
3-Bmi1 DR-GFP MOI10
4-Bmi1 DR-YFP MOI10
5-Ladder
6-Bmi1 DR-YFP MOI5

Figure S5. Full membrane exposition for Figures 2E and S2E, joined to the loading control.

Complete WB for Fig 3C

Exposition 1min, Anti-SV40 dil 1:100

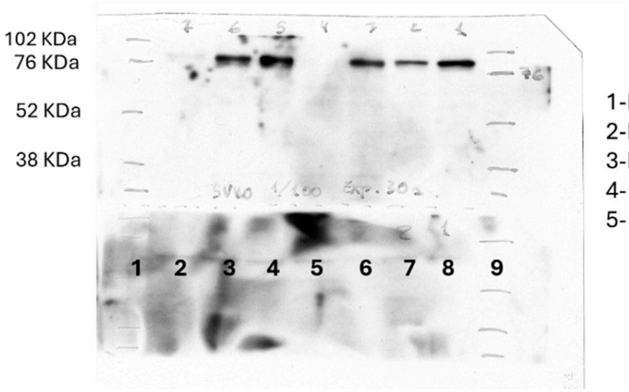

1-Ladder
2-Bmi1-DR$^{IMM-REV}$ + Adeno200
3-Bmi1-DR$^{IMM-REV}$ + Adeno100
4- Control Bmi1-DR^{IMM}
5- Bmi1-DR$^{IMM-REV}$ + Adeno200 + GCV

Exposition 1min, Anti-αTubuline dil 1:1000

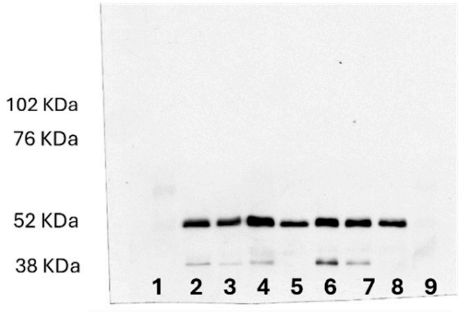

1-Ladder
2-Bmi1-DR$^{IMM-REV}$ + Adeno200
3-Bmi1-DR$^{IMM-REV}$ + Adeno100
4- Control Bmi1-DR^{IMM}
5- Bmi1-DR$^{IMM-REV}$ + Adeno200 + GCV

Figure S6. Full membrane exposition for Figures 3C, combined with the loading control.

Supplementary Tables

Supplementary Table S1: Primers Sequences.

Target Gene	Forward (5´- 3´)	Reverse (5´- 3´)
**GusB**	ACTCCTCACTGAACATGCGA	ATAAGACGCATCAGAAGCCG
**T-SV40**	GGTGGGTTAAAGGAGCATGA	AGGCTATCAACCCGCTTTTT
**TK**	CTCACCCTCATCTTCGACCG	CCTGCAGATACCGCACCGTA
**Bmi1**	CAGCAATGACTGTGATGC	CTCCAGCATTCGTCAGTC
**Cxcl12**	TTTCAGATGCTTGACGTTGG	GCGCTCTGCATCAGTGAC
**Cxcr4**	ACTCACACTGATCGGTTCCA	AGGTGCAGGTAGCAGTGACC
**VegfA**	AATGCTTTCTCCGCTCTGAA	GCTTCCTACAGCACAGCAGA
**VegfB**	GTGAAGCAGGGCCATAAAAG	GAGCTCAACCCAGACACCTG
**VegfC**	AGACGGACACACATGGAGGT	AAAGACTCAATGCATGCCAC
**VegfR1**	AAGAGAGTCTGGCCTGCTTG	CTGCTCGGGTGTCTGCTT
**VegfR3**	CCCCGGTGTCAATCACATA	CTCTGCCTCGGACTCCTC
**Pdgfra**	AGAAAATCCGATACCCGGAG	AGAGGAGGAGCTTGAGGGAG
**Cx43**	GGACCTTGTCCAGCAGCTT	TCCAAGGAGTTCCACCACTT
**Tpm1**	TCCAACTCCTCCTCAACCAG	CTCCGAGGCTCTCAAAGATG
**Bcl2**	ACATCCCAGCTTCACATAACCC	CCATCCCGAAAGAGTTCATTCAC
**Cat**	CCCCTATTGCCGTTCGATTCT	TTCAGGTGAGTCTGTGGGTTT
**Gpx1**	*CGCTTTCGTACCATCGACATC*	GGGCCGCCTTAGGAGTTG
**Prdx1**	GCCGCTCTGTGGATGAGATTA	AGCTGGACACACTTCACCAT
**Prdx4**	AGAGGAGTGCCACTTCTACG	GGAAATCTTCGCTTTGCTTAGGT
**Hmox1**	CACAGATGGCGTCACTTCGTC	GTGAGGACCCACTGGAGGAG
**EphB4**	GCCATCAAGATGGAAGATA	CACACTGGCCAAGATTTTCT
**EfnB2**	CCCTTTGTGAAGCCAAATCCAGGT	TCCTGATGCGATCCCTGCGAATAA
**Atg5**	AACCCGACCGAGCGGCTTTC	CAACCAAAGCCAAACCGAGGTGC
**Atg7**	TCTGGGAAGCCATAAAGTCAGG	GCGAAGGTCAGGAGCAGAA
**Atg12**	GGCCTCGGAACAGTTGTTTA	CAGCACCGAAATGTCTCTGA
**Atg13**	GTGGGCACCCTCACTCTTTC	GGGATAGGGACGGTCAACAA
**Map1lc3a**	CGTCCTGGACAAGACCAAGT	ATTGCTGTCCCGAATGTCTC
**Map1lc3b**	CCCCACCAAGATCCCAGT	CGCTCATGTTCACGTGGT
**Beclin**	CTTGGAGGAGGAGAGGCTGA	TGTGGAAGGTGGCATTGAAG
**Bnip3**	TCCTGGGTAGAACTGCACTTC	GCTGGGCATCCAACAGTATTT
**Apellin**	GGCCTTCTCCGTCTTTGTCG	CCCTCTTGTGCTTCTATCTCTCC
**Ppargc1a**	AAAAGCTTGACTGGCGTCAT	TGTCACTCCATACAGAGTCTTGG
**Atp5j**	TTCGGTCAGCAGTCTCTGTG	GCCTGTCGCTTTGATTTGTA
**Mgn**	GGTGTGTAAGAGGAAGTCTGTG	TAGGCGCTCAATGTACTGGAT

Supplementary Table S2: Antibodies, dilution and references.

	Antibody	Dilution	Comercial	Reference
**Western Blot**	αTubulina	1/ 1000	Calbiochem	CP06-100UG
	SV40 Tlarge	1/ 100	Calbiochem	DP02
	Ab Polyclonal Rabbit-HPRT Anti-Mouse IgG	1/ 1000	Dako Denmark	P0260
**FACS**	Sca1-PE	1/100	BD Pharmingen	553336
	cKit-PE	1/100	Beckman	734243
	Control Isotype Rat IgG2a-PE	1/100	Southern	0117-09
	CD45	1/100	BD Pharmingen	30-F11
	Control Isotype Rat IgG2b	1/100	Inmunotech	PNIM3040
	CD31	1/50	BD Pharmingen	553370
	PDGFRα	1/50	eBioscience	14-1401-82
	Control Isotype Rat IgG2a	1/50	Serotech	MCA1212
	Ab 2ª Goat Anti-Rat IgG2-PE	1/50	Southern	3052-9

Supplementary Table S3: **Tested factors and references.**

Factor	Reference
**CHIR99021**	*Sigma*; *SML1046*
**PD184352**	*Sigma*; *PZ0162*
**SU5402**	*Sigma*; *SML0443*
**BMP4**	*Sigma*; *H4916*
**Activin A**	*Sigma*; *SRP6153*
**N2**	*Gibco*; *17502-048*
**B27**	*Gibco*; *17504-044*

Supplementary Table S4: **Medium composition and references.**

Factors / Medium	LIF 10^3 U/mL	FBS	EGF 10 ng/ml	FGF 20 ng/ml	CHIR 99021 3µM	PD 184352 3µM	SU 5402 2µM	BMP4 (5 ng/ml) + ActivinA (10ng/ml) + N2 + B27
**Bmi1+DR**	+	+	+	+				
**Lalit et al.**	+	+			+			
**Zhang et al.**					+		+	+
**2i**		+			+	+		
**2i + LIF**	+	+			+	+		
**2ia + LIF**	+	+			+		+	

Supplementary Table S5: Tested inhibitors, concentration and references.

Inhibitor	Concentration	Target	Reference
**Rapamycin**	20 nM	*mTOR*	*Sigma*; *R0395*
**Pik75**	1 µM	*p110αβ PI3-Kinase*	*Selleckchem*; *S1205*
**TGX221**	40 nM	*p110β PI3-Kinase*	*Selleckchem; S1169*
**SB203580**	10 µM	*p38 MAP-Kinase*	*Selleckchem*; *Ref. S1076*
**BIRB796**	2,5 µM	*p38 MAP-Kinase*	*Selleckchem*; *S1574*
JAK 2 Inhibitor	40 µM	*JAK2*	*Cal Biochem, 420132*
**Y27632**	0,02 mM	*ROCK1 (p160ROCK)*	*Selleckchem*; *Ref. S1049*
